# Mapping the Structural and Dynamical Features of Kinesin Motor Domains

**DOI:** 10.1371/journal.pcbi.1003329

**Published:** 2013-11-07

**Authors:** Guido Scarabelli, Barry J. Grant

**Affiliations:** Department of Computational Medicine and Bioinformatics, University of Michigan, Ann Arbor, Michigan, United States of America; University of Illinois, United States of America

## Abstract

Kinesin motor proteins drive intracellular transport by coupling ATP hydrolysis to conformational changes that mediate directed movement along microtubules. Characterizing these distinct conformations and their interconversion mechanism is essential to determining an atomic-level model of kinesin action. Here we report a comprehensive principal component analysis of 114 experimental structures along with the results of conventional and accelerated molecular dynamics simulations that together map the structural dynamics of the kinesin motor domain. All experimental structures were found to reside in one of three distinct conformational clusters (ATP-like, ADP-like and Eg5 inhibitor-bound). These groups differ in the orientation of key functional elements, most notably the microtubule binding α4–α5, loop8 subdomain and α2b-β4-β6-β7 motor domain tip. Group membership was found not to correlate with the nature of the bound nucleotide in a given structure. However, groupings were coincident with distinct neck-linker orientations. Accelerated molecular dynamics simulations of ATP, ADP and nucleotide free Eg5 indicate that all three nucleotide states could sample the major crystallographically observed conformations. Differences in the dynamic coupling of distal sites were also evident. In multiple ATP bound simulations, the neck-linker, loop8 and the α4–α5 subdomain display correlated motions that are absent in ADP bound simulations. Further dissection of these couplings provides evidence for a network of dynamic communication between the active site, microtubule-binding interface and neck-linker via loop7 and loop13. Additional simulations indicate that the mutations G325A and G326A in loop13 reduce the flexibility of these regions and disrupt their couplings. Our combined results indicate that the reported ATP and ADP-like conformations of kinesin are intrinsically accessible regardless of nucleotide state and support a model where neck-linker docking leads to a tighter coupling of the microtubule and nucleotide binding regions. Furthermore, simulations highlight sites critical for large-scale conformational changes and the allosteric coupling between distal functional sites.

## Author Summary

Kinesin motor proteins transport cargo along microtubule tracks to support essential cellular functions including cell growth, axonal signaling and the separation of chromosomes during cell division. All kinesins contain one or more conserved motor domains that modulate binding and movement along microtubules via cycles of ATP hydrolysis. Important conformational transitions occurring during this cycle have been characterized with extensive crystallographic studies. However, the link between the observed conformations and the mechanisms involved in conformational change and microtubule interaction modulation remain unclear. Here we describe a comprehensive principal component analysis of available motor domain crystallographic structures supplemented with extensive unbiased conventional and accelerated molecular dynamics simulations that together characterize the response of kinesin motor domains to ATP binding and hydrolysis. Our studies reveal atomic details of conformational transitions, as well as novel nucleotide-dependent dynamical couplings, of distal regions and residues potentially important for the allosteric link between nucleotide and microtubule binding sites.

## Introduction

Kinesins are a large family of ATP-dependent molecular motor proteins that drive intracellular transport along microtubules. Family members have been found in all eukaryotic organisms, where they contribute to the transport of organelles, organization and maintenance of the cytoskeleton, and the segregation of genetic material during mitosis and meiosis. All family members contain one or more conserved motor domains that are responsible for both nucleotide and microtubule binding. Cycles of ATP binding and hydrolysis in these domains are coupled to a sequence of conformational changes that collectively generate force and movement along microtubules. Characterizing distinct motor domain conformations and their interconversion mechanism is thus essential to determining an atomic-level model of kinesin action.

**Figure 1:**
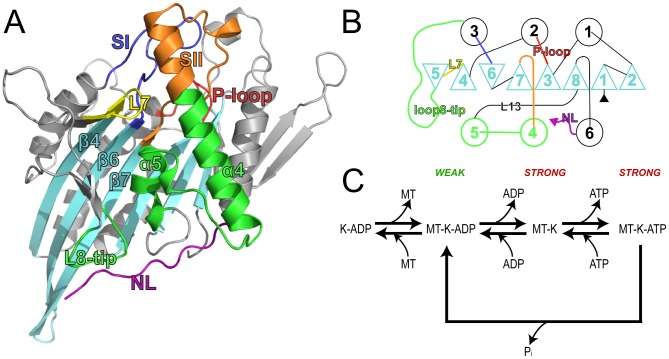
Structure and catalytic cycle of the kinesin motor domain. (**A**) The motor domain is composed of an eight-stranded antiparallel β-sheet surrounded by three major helices on either side. The ATPase catalytic site sits at the top of the β-sheet and is flanked by highly conserved loops, the P-loop (red), SI (blue) and SII (orange), which connect to helices on either side of the central sheet (α2, α3 and α4 respectively). The microtubule-binding interface has been mapped by Alanine scanning mutagenesis and limited proteolysis to the opposite side of the motor domain (green, encompassing the loop11, α4, loop12 and loop8 regions). Adjacent to the motor domain is the neck linker (purple), a flexible region that has been shown to undergo a nucleotide-dependent transition from a disordered to an ordered structure. Loop7 (yellow) and the motor domain tip are also indicated. (**B**) Motor domain secondary structure topology. β-strands are depicted as triangles and α-helices as circles. Regions are colored as in panel **A**. (**C**) Kinesin catalytic cycle. Kinesin (K) has a weak affinity for the microtubule (MT) in the ADP-state. ADP release, which is promoted by MT binding, is followed by strong binding to the MT. Subsequently, ATP binding may occur followed by hydrolysis and product release to regenerate the weakly bound ADP state.

Crystallography has yielded an abundance of high-resolution motor domain structures that provide valuable insight into critical structural variations (see Table S1). Analysis of these structures shows that conformational differences are largely concentrated in a small number of functionally important regions (Figure 1) [1]–[3]. These include local changes at the nucleotide-binding site, most notably in two conserved ‘switch loop’ regions (termed SI and SII or L9 and loop11, residues 222 to 235 and 266 to 287 in kinesin Eg5). Larger variations occur at the putative microtubule binding regions, loop8-β5 (residues 171 to 215) and α4-loop12-α5 (residues 288 to 327) and these changes couple to an order to disorder transition of the conformationally variable neck-linker region (residues 358 to 369). The neck-linker docking site spans the MT-binding interface. It joins to the neck helix α7 responsible for coiled-coil dimerization in a number of families (Figure 1). Recent Cryo-EM, EPR and FRET results have suggested that for kinesin I the neck-linker region is docked in the ATP state, whereas it is undocked and mobile in the ADP state [4], [5]. Together, these results have led to a general model for the transmission of structural changes from the nucleotide-binding site, through the microtubule-binding site, to the neck linker region [6]–[8]. However the detailed sequence of events and the mechanism by which the microtubule and nucleotide binding sites couple key dynamic rearrangements remains controversial.

The present study employs computational approaches to quantitatively assess the structural and dynamical features of distinct motor domain conformations and probe the apparent coupling between functional sites. Previous computational studies have provided considerable insights into kinesin dynamics. These include early studies, using simulated annealing [9] and free energy Poisson-Boltzmann surface area methods [10], that probed the mechanism of active site closure upon ATP binding. As well as more recent studies, utilizing elastic network normal mode analysis [11], coarse-grained [12]–[14] and [15] and targeted molecular dynamics [16], which have focused on structural variations in the nucleotide binding pocket and on the mechanism of neck-linker docking. From these, several residues have been identified as important for the underlying conformational mechanisms or kinesin microtubule interactions [16]–[19]. Most recently MD simulations of kinesin family member Eg5 successfully characterized fluctuations within individual nucleotide states but did not characterize significant interconversion events that are likely beyond the accessible nanosecond timescale [20]. These findings highlight both the utility of these approaches but also the need for new simulation approaches to probe functional transitions of not just the nucleotide binding-site or just the neck-linker but the entire motor domain.

A number of methods have been developed to enhance the sampling of slow conformational changes including conformational flooding [21], metadynamics [22] and accelerated molecular dynamics [23] (aMD). Within the framework of this study, a key advantage of aMD is that it allows us to study the conformational behavior and dynamics of the kinesin motor domain at full atomic resolution without using a pre-defined reaction coordinate. In previous studies, aMD has been successfully employed to study slow time-scale dynamics in HIV-protease [24], ubiquitin [25], and, of most relevance to the current study, the G-proteins Ras and Rho [26], [27]. In these last examples, aMD permitted the characterization of spontaneous nucleotide-dependent conformational transitions similar to those thought to occur in kinesin [6], [28].

In the following sections we examine the different crystallographic conformers and their relationship to those sampled by aMD simulations. The dynamics of ATP, ADP and nucleotide free kinesin are investigated and correlated motions that could be of functional importance are highlighted. The analysis reveals a good correspondence between the simulation results and the crystallographic structures. Additionally, the simulations provide new evidence for a dynamic linkage between functional sites that is not directly evident from the distribution of X-ray structures.

## Results/Discussion

We first mapped the distinct conformations of available experimental structures with principal component analysis (PCA). Accelerated molecular dynamics (aMD) simulations were then performed to characterize the intrinsic dynamics and response to nucleotide binding. Conformational sampling in these simulations was assessed by comparison with crystallographic results. Spontaneous transitions between major conformations and correlated motions were further analyzed to decipher important structural and dynamic features of potential functional relevance. Finally, *in-silico* mutations were employed to further investigate the importance of highlighted sites.

### Mapping distinct motor domain conformations

**Figure 2:**
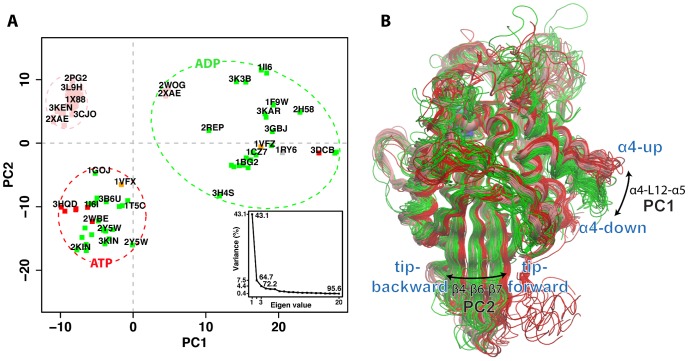
Results of PCA on the kinesin motor domain. (**A**) Conformer plot: projection of all kinesin X-ray structures onto the principal planes defined by the two most significant PCs (PC1 and PC2). Structures are colored by ligand bound, triphosphate (red), diphosphate (green), and Eg5 inhibitor (pink). Structures are also labeled with their RCSB PDB code where space permits (see Table S1 for full details). Colored dashed ovals represent the major groupings obtained from hierarchical clustering of the projected structures in the PC1 to PC5 planes (see cluster dendrogram in Figure S1B). Insert: eigenvalue spectrum detailing results obtained from diagonalization of the atomic displacement correlation matrix of Ca atom coordinates. The magnitude of each eigenvalue is expressed as the percentage of the total variance (mean-square fluctuation) captured by the corresponding eigenvector. Labels beside each point indicate the cumulative sum of the total variance accounted for in all preceding eigenvectors. (**B**) Kinesin motor domain structures colored according to their clustering in PC-space (i.e ovals in panel A). Red = ATP-like, Pink = Eg5 inhibitor-bound, green = ADP-like.

In total, 114 motor domain chains abstracted from 69 kinesin family crystal structures deposited in the RCSB protein data bank [29] (Berman et al., 2002) were subjected to interconformer analysis with PCA (see Table S1 and Methods for details). PCA was used to examine the major conformational differences between structures. Over 80% of the total mean-square displacement (or variance) of atom positional fluctuations was captured in five dimensions, over 72.2% in three dimensions, and over 64.7% in two dimensions. The first few PCs retain most of the variance in the original distribution, and thus provide a useful description of the conformational space of the system (see Figure 2 for details). Projecting the original structures onto the subspace defined by the PCs with the greatest associated variance resulted in a low dimensional, graphical representation that succinctly displays the relationship between structures (Figure 2A and S1).

Figure 2A displays the relationship between structures in terms of the conformational differences described by the first two PCs (PC1 and PC2). The contribution of each residue to these dominant PCs is displayed in Figure S2. The major feature described by PC1 is the concerted displacement of the regions comprising the consecutive secondary structure elements α4-loop12 (residues 291 to 305) and α5-loop13 (residues 308 to 315, 318, 321 to 329). This region comprises a mobile subdomain displaying the largest-scale concerted conformational displacements in the distribution of experimental structures. Positions within this subdomain are known to be a major component of kinesins microtubule binding site [30], . PC2 also demonstrates the inherent mobility of these regions, along with significant displacements of α2b-β4 and loop8 N-terminal (residues 136 to 148,152 to 159 and 170–173), β5-α3 (residues 199 to 204 and 205 to 219), β6 C-terminal and β7 N-terminal (residues 241 to 245 and 256 to 260). This portion of the central β-sheet (located at the bottom tip of the motor domain with respect to Figure 1) can adopt distinct ATP and ADP conformations (Figure 2B). It is also notable that distinct neck-linker orientations (docked, partially docked and absent) are clearly distinguished and coincident with the large-scale collective motions described by PC1 and PC2 (Figure S1). Structures showing α4 in the “up” orientation together with β4-β6-β7 positioned close to the neck-linker docking site (referred to as “tip-forward” in Figure 2B) have a fully-docked neck-linker, while the α4 “down” conformation and the beta strands of the motor tip close to α2 (referred to as “tip-back” in Figure 2B) correspond to an undocked or absent neck-linker. Interestingly, the Eg5 inhibitor-bound group shows α4-up but also β4-β6-β7 close to α2. In these structures, the neck-linker is partially docked with its N-terminal portion docked to the motor domain (but not the C-terminal) or it is completely absent.

**Figure 3:**
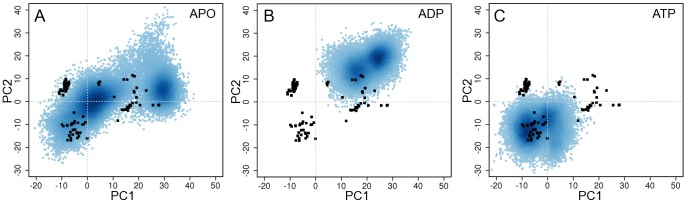
Analysis of structures from aMD simulations of Eg5. Projection of simulation snapshots sampled every 20ps from (**A**) nucleotide free, (**B**) ADP, and (**C**) ATP simulations onto the first two PCs defined by the X-ray structures (black circles, see Figure 2 and main text).

RMSD clustering is also consistent with the segregation of ATP- and ADP-like conformations (Figure 3S). However, what PCA explicitly adds is a cleaner separation (being less susceptible to displacements of isolated loops) and a dissection of how the structures collectively differ. Indeed, PCA clearly highlights that ATP- and ADP-like conformations are not only different in the microtubule binding site region (PC1) but also their tip β-sheet conformation (PC2). Collectively PC1 and PC2 separate ATP (α4-up, neck-linker docked, β-sheet tip back) from ADP (α4-down, neck-linker undocked, β-sheet tip forward) and inhibitor bound structures (α4-up, neck-linker partially docked, tip forward), see Figure 2B. The presence of an inhibitor molecule bound to the loop5 region in Eg5 subfamily members apparently stabilizes α4 in the ATP-like up-conformation, but at the same time β4-β6-β7 are shifted away from neck-linker binding site, assuming an orientation similar to the ADP-like structures. This suggests that the inhibitor decouples one of the two major conformational changes leading to an “up” α4 region (that favors N-terminal neck-linker docking) but ADP-like β-sheet tip (disfavoring C-terminal neck-linker docking).

The common conformational clustering obtained from RMSD and PCA do not coincide with the nature of the bound nucleotide in the various structures (point color in Figure 2A). This result is reminiscent of that obtained for F_1_ATPase [32] and myosin [27] where a strict one-to-one correlation between bound nucleotide species and global conformation is also not observed. In contrast, related monomeric and heterotrimeric G proteins were found to largely cluster into distinct triphosphate and diphosphate associated conformational groups [27], [33], [34]. Indeed, aMD simulation studies of these systems have indicated that the conformational variations characterizing different Ras, Rho and transducin G protein structures are intrinsically accessible to the corresponding nucleotide free protein [27], [33], [34]. Whilst in the presence of GDP or GTP, simulations under the same conditions resulted in restricted sampling in regions around the corresponding cluster of GDP or GTP crystallographic structures [27]. These results suggest a mechanism involving nucleotide induced conformational selection from a preexisting equilibrium distribution of conformers that includes both diphosphate- and triphosphate-like conformations [35]. The current analysis indicates that available kinesin experimental structures do not follow the G protein trend of displaying a relatively tight correlation between nucleotide state and global conformation. However, it is conceivable that in the presence of microtubules, distinct nucleotide state associated kinesin conformations may become more apparent, as suggested by others [7], [15], [36]. In this scenario interaction with microtubules would lead to a tighter coordination of nucleotide state and global motor domain conformation. Below we describe the results of aMD simulations that further probe the apparent susceptibility of kinesin, in the absence of microtubules, to assume multiple conformational states regardless of the bound nucleotide.

### Characterizing motor domain dynamics with aMD

Duplicate 200ns aMD simulations were performed to further characterize the intrinsic dynamics of ATP, ADP and nucleotide free Eg5 kinesin motor domains (Table 1). In all simulations, the core residues of the motor domain (comprising the upper portion of the central beta sheet and P-loop region, see Methods) exhibited relatively low RMSD fluctuations (mean value of 0.90±0.28 Å), indicating that the core structure of the protein is closely maintained throughout all simulations (Figure S4). The high RMSD values obtained for the entire motor domain (*e.g.* mean value of 3.12±0.46 Å for nucleotide free simulations) are similar in magnitude to the difference between the various crystal structures (*e.g.* 3.54 Å between kinesin 1, PDB code: 2KIN, and kinesin 14, PDB code 3KAR). Notably larger fluctuations were observed for the nucleotide free and ADP simulations (3.12±0.46 Å, 3.09±0.69 Å, and 2.58±0.38 Å for APO, ADP and ATP respectively) indicating enhanced rigidity of the ATP bound system. The structural flexibility at each position in the motor domain was assessed by comparing the atomic positional fluctuations calculated from simulations to both crystallographic B-factors and the atomic fluctuations derived from the large distribution of available X-ray structures. A good correspondence between experimental structure deviations and the average fluctuations calculated from each of the MD simulations was obtained (R^2^ 0.83). Furthermore, each simulation was found to exhibit a common dynamic trend consistent with the trend of differences between X-ray structures (Figure S5). The predominant dynamic signature consists of rigidity in the top portion of the central β-sheet (the structural core), with higher mobility at the motor domain tip and in the surrounding α-helices and surface exposed loops. The largest fluctuations were found to occur in loop2, loop5, loop9, loop10, loop11 and the C-terminal neck-linker (discussed later). The general agreement between the different simulations, B-factors and the fluctuations computed from the distribution of available X-ray structures indicates that the relative magnitude of displacements for individual residues is being simulated accurately.

**Table 1:** Simulations performed.

System	Simulation Time (ns)	RMSD (Å)
Eg5-APO	200×2	2.54±0.42 ; 3.45±0.88
Eg5-ADP	200×2	2.23±0.26 ; 2.43±0.30
Eg5-ATP	200×2	2.00±0.37 ; 1.99±0.45
Eg5-G325A/G326A-APO	100×2	1.93±0.24 ; 1.92±0.30

Simulations were run in pairs, differing only in the assignment of initial velocities, to improve sampling [33], [49]. RMSD values averaged over production phase dynamics for each simulation were calculated based on the Cα atom subset used for experimental structure PCA (see Methods).

Projection of the simulation conformers onto the PCs determined from analysis of kinesin crystal structures (see previous section) was used to evaluate the overall conformational space sampled by each set of simulations (Figure 3). It is apparent from these projections that each set of simulations sampled a wide region of conformational space, but that nucleotide free systems covered a much wider region than ADP or ATP simulations. This result indicates that the main collective displacements evident in the large distribution of crystal structures are accessible during aMD simulations of nucleotide free motor domains. These APO simulations sampled two distinct regions: one near the cluster containing the ATP-like crystal structures, and another close to the cluster of ADP-like structures, suggesting a spontaneous ATP-to-ADP transition. Indeed the conformations of the α4 subdomain and α3-loop8 together with β4-β6-β7 regions undergo large-scale structural changes, visiting a range of different orientations shown in the crystal structure ensemble (discussed further below). In contrast, ADP and ATP simulations display a more restricted sampling (Figure 3B & C) with overall smaller deviations for both the α4 subdomain, nucleotide binding site and the motor domain tip regions (Figure S5). This finding indicates that the ligand has an active effect on the flexibility of these regions resulting from direct structural contacts with loop9 and loop11. Furthermore these results support the notion that the structural change from ADP-like to ATP-like forms evident in the crystallographic dataset is already encoded in the intrinsic dynamics of the nucleotide free motor domain with nucleotide enhancing the rigidity of the system and effectively narrowing conformational spread. In an analogous proposal Jana *et al.* have recently reported, using a dual-basin structure based model, that microtubule binding can reduce the configuration space of the Ncd motor domain relative to the unbound form [15].

Although nucleotide free simulations display a comparatively higher degree of flexibility we do not observe evidence for substantial unfolding events: As assessed from measurements of the radius of gyration, overall secondary structure content, core residue RMSD and visual inspection (note that values for these metrics are similar to those obtained for the distribution of crystal structures and ADP and ATP bound simulations (see supplementary Table S2). Furthermore, the conformations sampled during nucleotide free simulations are similar to those observed for both ADP- and ATP-like crystal structures (see Figure 3). We also note the availability of a nucleotide free kinesin experimental structure (PDB code: 1RY6) that has a similar overall secondary structure content to that of other nucleotide bound structures as well as the experimentally demonstrated stability of nucleotide free Eg5 by Cochran and Gilbert [37]. Further support for the relevance of the heightened flexibility observed in the current nucleotide free simulations stems from the analogous behavior noted previously for the related Ras, Rho and heterotrimeric G proteins [27], [34]. In these cases the nucleotide free states also displayed the largest degree of flexibility but were observed to maintain their overall folded form.

### Large scale motions of dynamic subdomains

**Figure 4:**
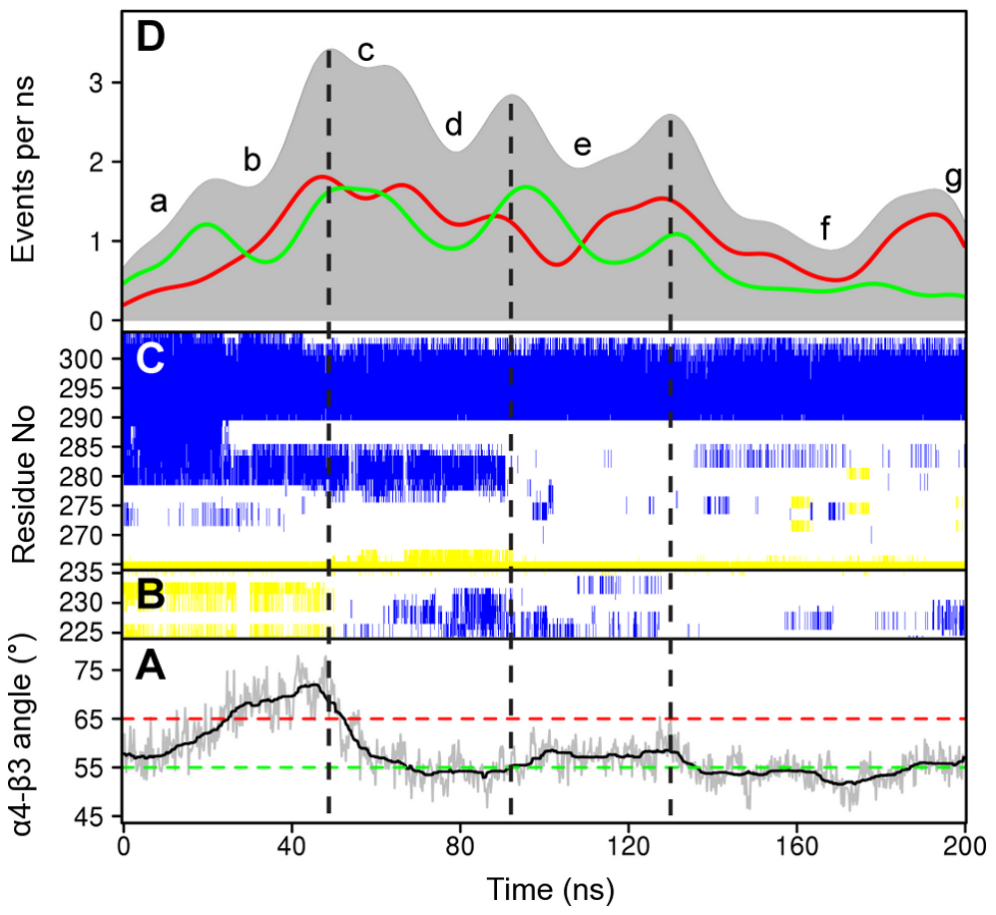
Time evolution of local and global structural changes. (**A**) The protrusion angle of α4 relative to β3. Dashed lines depict the average angle for ATP-like (red) and ADP-like (green) crystallographic structures. (**B**) The secondary structure content of SI and (**C**) SII-α4. Yellow represents beta strand, blue alpha helix, and white coil secondary structure types (see text for details). (**D**) Contact formation and breaking activity during nucleotide free simulations. The plot reports the contact formation events (green), the contact breaking events (red) and total events (gray, formation + breaking) as a function of simulation time.

Performing PCA on conformers derived from the simulations indicated that the eigenvectors extracted from the crystal structure distribution are also among the most dominant collective motions explored in the simulations (Figure S6). The major collective motion in all simulations involves α4 subdomain re-orientation. Monitoring the relative angle and distance between the center of mass of α4 with that of β3 in the structurally invariant core characterizes the protrusion of the α4 subdomain from the core of the fold. On average, the α4 “up” conformation displays a distance value of 16 Å and angle of 65^0^. In all simulations, α4 reduces its protrusion and moves closer to the core (for nucleotide free simulations the distance drops to ∼13 Å with a relative angle below ∼55^0^, Figure 4A). Consistent with these measures ADP-like crystal structures are characterized by 13 Å and 55^0^ degree α4–β3 orientations. PCA on the nucleotide free trajectories also highlight the collective motion of the motor domain tip regions. Indeed those movements dominate the second eigenvector (Figure S2). This motion is not apparent in the ATP and ADP simulations. Hence the absence of nucleotide results in an increased flexibility of these elements facilitating their movement.

### The dynamics of the nucleotide binding site and neck linker regions

Notable conformational transitions are apparent for two nucleotide binding site loops (SI and SII) and the neck-linker region that connects the motor domain to the N-terminal of the coiled coil dimerization domain. In the crystallographic dataset SI is present as a two-stranded beta sheet (e.g. PDB code: 3HQD), a short alpha helix (PDB code: 1II6 or random coil structure (PDB code: 2ZFJ). In the nucleotide free simulations this region samples each of these secondary structure types (Figure 4B). In the absence of nucleotide SI was observed to change from its initial beta strand structure to an alpha helix (∼50 ns) and then random coil (∼110 ns). In contrast this region maintains a helical structure in ADP simulations and beta strand in ATP simulations. The increased mobility of SI in the absence of nucleotide is consistent with a decrease or loss of the electron density of this region in the recently reported EM structure of nucleotide-free kinesin [38]. The current simulations indicate that the gamma phosphate of ATP increase the stability of the SI beta strand conformation of this intrinsically variable segment.

The SII loop11 region was observed to elongate in nucleotide free simulations due to the partial shortening of the N-terminal segment of α4 (up to the conserved Eg5 residue N289, Figure 4C). The unfolding of this segment begins at the N287-I288-N289 positions. The loss of helical structure in this region destabilizes the entire SII region and affects the interactions formed by the so called latch residues Y164 (β4/loop7) – R234(SI) – E284(SII) as well as the inter-switch salt bridge R234(SI) – E270(SII). A similar shortened α4 helix is observed in many of the available crystal structures (e.g. PDB code: 1BG2), indicating that this simulated change in protein structure is consistent with differences observed between independent experimental structures. The coil configuration of SII is highly flexible (with a mean RMSF of 4.28 Å) and can re-orient toward the groove between α4 and α6, or alternatively interact with the nucleotide binding regions, reforming the latch and salt bridge interactions. Previous work has indicated that the inter-switch salt bridge is likely required for hydrolysis-competence [39]. (Discussed further below).

Other important changes were evident for the neck-linker region. In the crystallographic dataset the neck-linker has been observed in a so called ‘zipped’ or docked conformation localized to the main body of the motor domain (and in some structures such as 3KIN participating in a beta sheet with the N-terminal cover strand, generating the so called ‘cover neck bundle’ [16]). Alternatively, the neck-linker can be undocked and displaced from the motor domain assuming a random coil structure. The current analysis of crystallographic structures indicates that both ADP and ATP analogue bound experimental structures display undocked neck-linker regions (detailed in Figure S1). However, there are a lower number of experimental structures with an undocked neck-linker in the ATP-like conformational group and only the ATP-like conformations display fully docked neck-linker regions for several structures (see Figure S1). This suggests that the neck-linker docked conformation is more favorable in the ATP-like conformational state, but that undocking is still physically possible. To analyze the possible undocking of the neck-linker in simulations the interactions formed by I359 with loop1 (N18, I19), α4 (I299, L302, V303) and loop13 (L324) were monitored. These interactions distinguish Eg5 experimental structures with docked neck-linker (e.g. PDB code: 3HQD) from those with undocked neck-linker (e.g. PDB code: 1II6). The breaking of these initial interactions and consequent neck-linker detachment were observed in all simulations (Figure S7). Thus the undocking of the neck-linker did not show a correlation with the ligand bound in the binding pocket of the various simulations. We note that the last residue present in our models (K368) is equivalent to the last residue observed in the neck-linker-docked Eg5 crystal structure 3HQD. This region encompasses the β9 segment of the neck linker but excludes a portion of the C-terminal β10. Experiments have noted that the docking of β9 to the catalytic core is the major contributor to the power stroke and increased velocity of Kif1A [40]. However, it is possible that the presence of additional N-terminal residues may alter this regions behavior. It is also possible that distinct kinesin subfamilies may display distinct neck-linker behaviors that could be related to their different functionalities. In this regard additional simulation data for a range of kinesin subfamiles should prove informative.

In summary, as previously shown for the α4 and α3 subdomains, the transitions of SI, SII and the neck-linker observed in the crystallographic dataset are intrinsically accessible to the nucleotide free motor domain in our simulations. Furthermore, for SII and the neck-linker regions there is no strict correlation between the conformation of these regions and the nature of the ligand present in the binding pocket. SI secondary structure instead is influenced by the ATP nucleotide, the third phosphate of the ligand forms an interaction with N229 promoting a beta strand conformation that is less stable in other systems.

### Contact event activity analysis

To further characterize potentially important structural transitions the time evolution of residue contacts was determined with the TimeScapes method [41]. This analysis monitors the formation and breaking of contacts between residues and reports an overall contact event activity level that can be used to identify significant conformational transitions (see methods). Figure 4 reports the results of this analysis on a representative nucleotide free simulation along with the corresponding structural variations of SI, SII and helix α4 regions. In addition to monitoring the time evolution of conformational transitions this analysis permitted the clustering of trajectory segments into regions with similar inter-residue contacts. This partitioning was found to be consistent with the results of RMSD clustering with the added advantage of being less sensitive to the motion of long surface loops such as loop11. For the trajectory in Figure 4 seven segments were apparent (labeled a-g in Figure 4D) and the activity curve clearly points out α4–α5 subdomain variations (see also Figure 4A). In the first segment, (a), α4 starts in a “down” orientation with an angle below 55^0^. In segment (b) the angle swings “up” to 75^0^ and then drops to 55^0^ again in segments (d) and (f) that are separated by another ATP-like “up” protruding orientation in segment (e). Both RMSD and distance in PC-space to the ADP- and ATP-like structures also decreases at these time points. These results together indicate that α4 can sample both ATP- and ADP-like configurations.

### Coupling of microtubule binding site, neck-linker and nucleotide binding site

**Figure 5:**
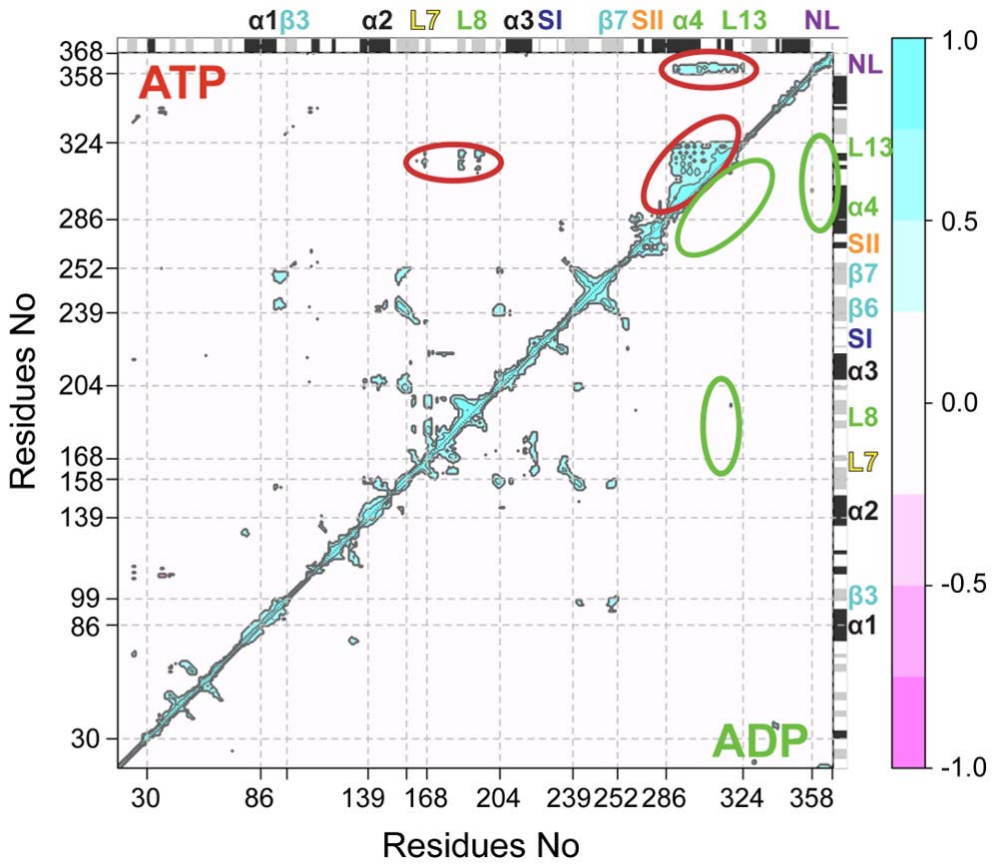
Residue-residue plot of correlated motions. The extent of correlation of atomic displacements for all residue pairs during ATP-like (upper triangle) and ADP-like (lower triangle) trajectory segments. The color scale runs from pink (for values ranging between −1 to −0.5), through white (−0.5 to 0.5) to cyan (0.5 to 1). Negative values are indicative of displacements along opposite directions, namely anticorrelated motions, whereas positive values depict correlated motions occurring along the same direction. Major secondary structure elements are labeled and indicated schematically with helices in black and strands in gray.

To examine whether the motions of one residue are related to the motions of another (distant) residue, the correlation of the displacements of all residue pairs were determined. As expected, in all cases the strongest positive correlations exist between covalently bonded residues and those residing within the same secondary structure elements (Figure 5). Moving up the diagonal, the first area of significant correlation corresponds to the α0-loop1-β1-loop2 region (residues 30 to 73). The consistent appearance of correlated motions for these residues in the simulation and the cross-correlation (off-diagonal peak) with β8 and loop14 (residues 328 to 335) further highlight the subdomain-like structure and dynamics of this portion of the motor domain. The second major region of note corresponds to the loop8-β5 structural elements (residues 168 to 204). This region has previously been identified as important for microtubule interaction [31], and will be discussed further below. The next major region of correlation corresponds to portions of the central beta strands β6–β7 (residues 240 to 249–253 to 262) together with α1b-β3 (residues 86 to 99), α2b-β4 and β5 (residues 139 to 158 and 200 to 204), (note the off-diagonal peaks between these regions). These structurally adjacent strands from the mobile tip of the motor domain display more persistent correlations with each other than to other residues in the upper core β-sheet and P-loop regions of the motor domain.

One of the most notable features of the plots is the pattern of correlation for the α4-loop12-α5-loop13 region (residues 286 to 324). Each simulation shows a high degree of correlation for residues residing within α4 and α5, consistent with its sub-domain dynamics noted earlier. However, the correlation of the entire region (including loop12 and loop13) is most apparent during ATP-like trajectory segments when cross-correlations are also evident for the loop8-β5 region and the neck-linker (residues 358 to 368, upper triangle). This result highlights a coupling between distal microtubule binding site elements and the neck-linker when the motor domain assumes an ATP-like global conformation. However, the actual residues critical for the coupling between the various regions cannot be easily identified using solely this analysis. To further dissect the apparent couplings we employed the dynamic network analysis methods developed by Luthey-Schulten and colleagues [42]. In this approach a weighted graph is constructed where each residue represents a node and the weight of the connection between nodes represents their respective correlation value. A clustering of edges is then used to define local communities of highly correlated residues that represent substructures that are highly intraconnected, but loosely interconnected (see Methods). By definition, nodes in the same community can communicate with one another relatively easily through multiple routes. However, there are comparatively few edges involved in communication between communities, and the residues involved in this inter-community communication have been shown to be important for allosteric signaling in a number of systems [43], [44].

**Figure 6:**
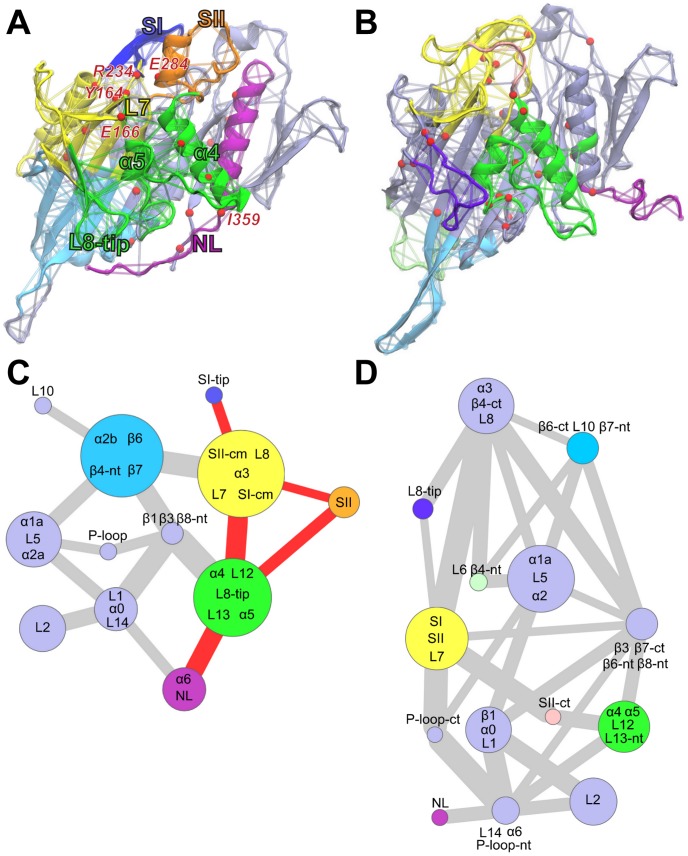
Dynamically coupled networks for ATP-like and ADP-like conformations. Molecular figures (**A** and **B**) highlighting communities (composed of clustered edges – representing residues) for ATP and ADP states respectively. Red spheres indicate residues that occur in a majority of shortest paths connecting nodes in different communities (i.e. residues important for the connection between communities). (**C** and **D**) Community network representation (colored as in **A** and **B**). Each node (circle) represents a community with its grouped protein regions labeled. The width of connecting lines is proportional to the number of shortest paths passing through corresponding junctions (i.e. their betweenness values). The pathway connecting nucleotide binding switch regions to the neck-linker in the ATP-like conformation graph is highlighted in red. Abbreviations: L = loop, cm = conserved motif, nt = n-terminal, ct = c-terminal.

Applying this method to kinesin indicates that, for ATP-like conformations, a large community is formed by α4-loop12-α5-loop13 (N289-R327), loop8 (M184-I195) and loop7 (E166) regions (Figure 6A and 6C). Portions of loop7 are also dynamically coupled to the nucleotide-binding SI and SII regions as discussed below. Remarkably, loop13 shows connections to the adjacent α5 and α4 as well as the distal loop8, thus encompassing a large proportion of the likely microtubule-binding site. In contrast, for ADP-like states these regions form separated communities, indicating a lower degree of overall coordination consistent with the raw correlation analysis results (Figure 6B and 6D). It is notable that a consistent dynamic community partitioning for these regions was observed in sets of eight 40 ns ATP and ADP conventional MD (cMD) simulations each performed with different initial conditions (see Figure S8 and Table S3). Furthermore, a pathway of interlinked dynamic residues connecting the nucleotide-binding, microtubule-binding and neck-linker regions is clearly apparent in the ATP simulation set. This coupling involves conserved residues from SI, SII, loop7, α4, α5, loop8 and the N-terminal portion of the neck-linker. The emergence of these couplings in the ATP-like portions of aMD simulations and in multiple independent cMD simulations (see Figure S8) suggests the presence of a conformationally dependent communication flow between the nucleotide-binding, microtubule-binding and neck-linker regions through loop7. In contrast, evidence for this complete pathway in multiple ADP set simulations is not observed due to the general lower coordination among these regions.

Conserved residues from the nucleotide binding SI and SII motifs are consistently grouped together with residues in loop7 in both ATP and ADP set simulations. In ATP-like configurations, the pathways connecting the nucleotide-binding site and the microtubule-binding site communities involve an extend set of well-conserved residues in these regions (see Table S4). Most notably the SI residues R234 and S235 coordinate with loop7 Y164, while loop7 E166 and E167 emerge as important for the connection to the microtubule binding site communities containing loop8 and α4–α5. The N-terminal neck-linker residue I359 and neighboring residues are highlighted as critical for the interactions with adjacent residues in the microtubule-binding site. This observation is in agreement with experimental data reporting that mutations of the N-terminal portion of the neck-linker, including I359, has a larger influence on functional properties than mutations to C-terminal neck-linker residues [45]. Additional mutational studies of Kar3 have indicated R234 plays a role in linking microtubule binding to the resulting stimulation of ATPase activity [39]. The current results indicate that mutations of E166-E167 may also disrupt the allosteric link between microtubule and nucleotide-binding sites. Indeed, equivalent residues to E166 and E167 have been shown to be important for Kif1A microtubule interaction [40]. Nitta and coworkers have also described the so-called “latch interaction” among residues equivalent to those highlighted in this study, namely Y150, R216 and E267 of KifA [40]. These residues correspond to our highlighted Eg5 Y164 (loop7), R234 (SI) and E284 (SII). These interactions are observed to break and reform during all simulations regardless of the conformational variations in SI, SII and α4 regions. However, network analysis shows connections among these nodes in ADP-like conformations, which are absent in ATP-like states. Furthermore, the formation of the latch connections precedes the conformational transition to ADP-like states, supporting the idea that a change in the nucleotide-binding site may affect the microtubule-binding site, specifically α4 protrusion and neck-linker detachment, via the identified loop7 residues.

In summary, the correlation and dynamic network analyses suggest that there is coordination between different elements of the microtubule-binding interface in ATP-like states that is reduced or absent in ADP-like states. Variations of the angle and protrusion distance are associated to the correlations of the α4 subdomain with the neck-linker and with the microtubule binding site elements. Only with α4 protruding are these protein regions dynamically coupled. Residues in loop7 are highlighted as key for the dynamic transfer of information between sites. We speculate that the lack of coupling in ADP-like orientations may be related to the observation that the kinesin ADP-bound form is more weakly prone to interact with the microtubule than ATP or APO forms [46], [47]. The current dynamic coupling analysis provides evidence for, and the identities of, sites that link the dynamics of the microtubule-binding site to the nucleotide state of the system. In the next section we employ mutational analysis to further probe the significance of these couplings.

### *In silico* mutations disrupt coupling and are known to affect function

Our analysis has highlighted sites as potentially important for the conformational mechanism and allosteric coupling of distal sites in the kinesin motor domain. One such site is loop13 (positions 324 to 327), which is not likely to directly contact the microtubule but emerges as potentially important for coordinating loop8 with α4-loop12-α5 in ATP-like conformations. Furthermore, previous studies have indicated that mutations of two glycine residues in loop13 impairs the motility of kinesin-1 in gliding assays with little significant effect on the ATPase activity [45]. The focus of this experimental mutagenesis study was on the possible effects of these residues on neck-linker docking and not dynamic coupling. However, we note that, many other ATP and GTP binding proteins show two small non-polar residues (predominantly glycine or alanine) in an equivalent structural position to loop13 [Grant, in preparation], suggesting that this region might be important for the potentially conserved dynamics of these systems. In the current aMD simulations, a strong coordination among the complete α4 subdomain elements (α4, loop12, α5 and loop13) was detected with the neck-linker and with loop8 in ATP-like states. For these reasons, and to examine the structural and dynamic effects this portion has on the motor domain, two aMD simulations of 100 ns each in the nucleotide free state were performed on the Eg5 G325A-G326A double mutant.

**Figure 7:**
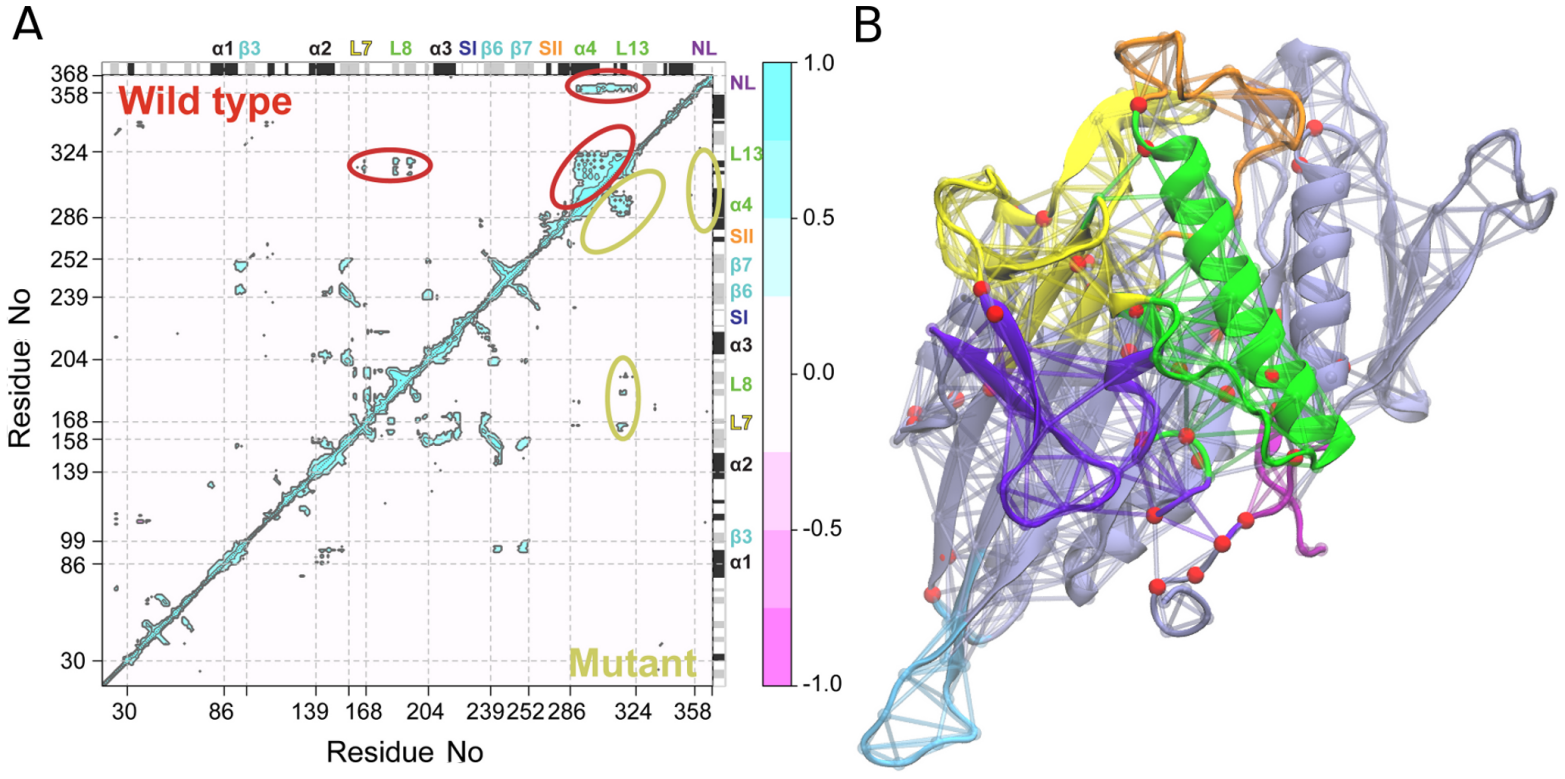
Results of loop13 G325A/G326A simulations. (**A**) Correlation of atomic displacement for all residue pairs for Eg5 mutant G325A/G326A. The color scale runs from pink (for values ranging between −1 to −0.5), through white (−0.5 to 0.5) to cyan (0.5 to 1). Negative values are indicative of displacements along opposite directions, namely anticorrelated motions, whereas positive values depict correlated motions occurring along the same direction. Major secondary structure elements are labeled and indicated schematically with helices in black and strands in gray. (**B**) Molecular representation of dynamic community partitioning for G325A/G326A mutant.

Analysis of the resulting trajectories showed a dramatically reduced flexibility of the protein with respect to wild-type (RMSD 2.26±0.32 Å vs 3.12±0.46 Å). Most notably the orientation of α4 does not change significantly, the neck linker does not detach and the key functional elements SI and SII remain stable in their initial beta-strand and alpha helix configurations. PCA analysis also indicates that the mutations impair the sampling of the ADP-like state evident in wild type nucleotide free simulations. In addition, correlation and dynamic network analysis of the mutant trajectories highlight a lower connectivity of loop13 to neighboring residues (Figure 7). Specifically, position G325A displays a reduced degree of dynamic coupling with other sites including α4, α5 and loop 8. This results in the clustering of loop13 into a separate community from α4 and α5 in all mutant simulations. Furthermore, even though during the simulations the motor domain samples ATP-like states (in close vicinity to the initial configuration), loop8 does not show any dynamic coupling to loop13, resulting in a reduced coordination of the microtubule-binding interface as evident with wild-type ADP-like conformations.

In summary, these results suggest an interpretation at the atomic level of the experimental data available on this mutant. The substitution of two glycine residues with alanine in loop13 reduces the overall flexibility of this region causing a decoupling in the coordination of the surrounding structural elements effectively hindering the reorientation of α4 and α5 and their coupling to the nucleotide-binding site.

### Conclusions

We have characterized the structural and dynamical features of the kinesin motor domain from analysis of available crystal structures and accelerated molecular dynamics simulations. Both experimental and simulated datasets identified the variation of the α4–α5 subdomain as the major large-scale collective motion. Indeed kinesin structures and trajectory segments can be classified in two distinct conformations based on the configuration of the α4–α5 subdomain. Structures showing a protruding α4 helix are defined as ATP-like states, while structures with reduced α4 protrusion are associated with ADP-like states. Intriguingly ATP and ADP like conformations are not directly correlated to the ligand bound in the active site, but rather are intrinsically accessible to the protein structure. The complete transition from ATP to ADP like configurations was observed in nucleotide free simulations. The structural variation of α4 was also evident in nucleotide bound simulations, again supporting the idea that the ligand bound does not directly dictate the global subdomain orientation. In addition, neck-linker, SI and SII regions show important variations. The neck-linker undocks and separates from the main body of the motor domain facilitating α4 movement. In the APO trajectories, SI samples all the conformations solved in the experimental ensemble, while the SII extended starting helix unfolds resulting in an elongated or shortened loop11, a feature also evident in the accumulated experimental structures.

Correlation and dynamic network analysis identified a cluster of residues formed by loop8, α4, loop12, α5, loop13 and the neck-linker as dynamically coordinated in ATP-like conformations. The neck-linker docked configuration favors α4 protrusion helping maintain the ATP-like state. In addition, the community clusters obtained with the dynamic network analysis further characterized the microtubule binding site and motor domain tip in the ATP and ADP-like conformations, highlighting residue connections and determining E166-E167, R234-E284-Y164 and I359 as key for the communication between nucleotide-binding, microtubule-binding and neck-linker sites. This newly predicted role for residues in loop7 is directly testable by mutagenesis studies. Additionally, simulations of the G325A/G326A mutant in loop13 highlighted the importance of these positions for the inherent flexibility of the α4 subdomain. Mutation of these sites perturbs the motion of the microtubule binding site regions (α4–α5 and loop8) and the extensive coupling of these sites evident in wild type systems. We anticipate that further mutational analyses of the positions identified here as potentially critical for allosteric coupling will help in characterizing the underlying mechanisms of kinesin function at the molecular level.

## Methods

Unless otherwise noted, the Bio3D package (version 1.1–4) was used for all analysis [48]. Atomic coordinates for all available kinesin crystal structures (87, as of July 2012, see Supplemental Table S1) were obtained from the RCSB Protein Data Bank [29]. A total of 18 structures were excluded from analysis due to absence of resolved residues in key nucleotide binding site regions. Prior to assessing the variability of the remaining 69 crystal structures, iterated rounds of structural superposition were used to identify the most structurally invariant region. This procedure entailed excluding those residues with the largest positional differences (measured as an ellipsoid of variance determined from the Cartesian coordinates of equivalent Cα atoms), before each round of superposition, until only the invariant “core” residues remained [48]. This structurally invariant core consists of 63 residues (encompassing portions of the structural elements β1, β2, β3, P-loop, α2a, β6, β7 and α6), and was used as the reference frame for superposition of both crystal structures and subsequent MD trajectory snapshots. Nucleotide annotations for each structure were derived from the HETATOM records of the original RCSB PDB files.

### Principal component analysis

Principal component analysis was used to characterize the relationships between superposed structures and molecular dynamics trajectory output. The application of PCA to both distributions of experimental structures and MD trajectories, along with its ability to provide considerable insight into the nature of conformational differences in a range of protein families has been previously discussed [3], [33], [49], [50]. Briefly, PCA is based on the diagonalization of the covariance matrix, *C*, with elements *C_ij_*, built from the Cartesian coordinates, *r*, of the superposed structures:
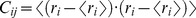
(1)where *i* and *j* represent all possible pairs of 3N Cartesian coordinates, where N is the number of atoms being considered. The eigenvectors of the covariance matrix correspond to a linear basis set of the distribution of structures, referred to as principal components (PCs), whereas the eigenvalues provide the variance of the distribution along the corresponding eigenvectors. Projecting structures into the sub-space defined by the largest principal components (along which the sample variance is largest) results in a lower dimensional representation of the structural dataset (see Figure 2 for details). The resulting low-dimensional ‘conformer plots’, succinctly display the major differences between structures, highlight relationships between different specific conformers and thus enable the interpretation and characterization of multiple inter-conformer relationships [48].

For the current application we included only those regions of structure for which equivalent residues are found in all structures. More specifically, the neck-linker region did not factor into our current PCA analysis due to its absence in a number of the available structures. However, a similar analysis that includes only the subset of structures for which the neck linker is present (and thus explicitly includes the neck-linker region) results in a similar pattern of overall inter-conformer relationships (Figure S1 and S9).

### Molecular dynamics simulations

Simulation models were based on the recent high-resolution structure of human Eg5 kinesin in complex with the non-hydrolyzable analogue AMPPNP (PDB code: 3HQD) and ADP (PDB code: 1II6). AMPPNP was modified to ATP or removed completely for the respective ATP and nucleotide free simulation starting models. The missing loop12 region in PDB code 1II6 was modeled with Modeller v9.10 [51] and evaluated with the DOPE score [52] to yield the ADP simulation starting model. Two accelerated molecular dynamics simulations were performed for each system using the AMBER11 package [53] and corresponding all-atom potential function ff99SB [54]. An additional eight conventional molecular dynamics simulations were performed for ATP and ADP systems. Operational parameters include periodic boundary conditions, TIP3P water and charge-neutralizing counter ions, with full particle-mesh Ewald electrostatics. A 2fs time step and a 10 Å cutoff were used for the truncation of VDW non-bonded interactions. Constant volume heating (to 300 K) was performed over 10ps, followed by constant temperature (300 K), constant pressure (1 atm) equilibration for an additional 200ps. Finally, constant pressure constant temperature production dynamics was performed. The SHAKE algorithm was used to constrain all covalent bonds involving hydrogen atoms. In order to simultaneously enhance the sampling of internal and diffusive degrees of freedom a dual boosting accelerated molecular dynamics (aMD) approach was employed, based on separate torsional and total boost potentials [55]. The energy level, E, below which the boost is applied and tuning parameter, α, that modulates the depth and local roughness of basins in the modified potential, were based on previous works [26], [27].

### Cross correlation and dynamical network analysis

To identify protein segments with correlated atomic motions the cross-correlation coefficient, *C_ij_*, for the displacement of all Cα atom pairs, *i* and *j*, was calculated:
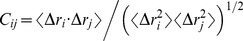
(2)where *Δr_i_* is the displacement from the mean position of the *ith* atom determined from all configurations in the trajectory segment being analyzed (see [56] and [57] for further details). Using these data dynamical networks were constructed following the method of Luthey-Schulten and colleagues [42]. In this approach a weighted graph is constructed where each residue represents a node. Two nodes are connected in the network if they are in contact during the trajectory segment under analysis; i.e., their closest heavy atoms are within 4.5 Å for 75% of simulation frames. Edges between nodes *i* and *j* are weighted (*w_ij_*) by their respective correlation value (*C_ij_*):
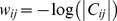
(3)


Hierarchical clustering was used to generate aggregate nodal clusters, or communities, that are highly correlated and within close physical proximity. Network analysis concepts (i.e., shortest path, centrality, and suboptimal path analysis) were used to identify prominent nodes and paths in the network using the VMD dynamical network analysis plugin [58].

### Contact activity analysis

Analysis of rare contact formation and breaking events associated with conformational changes was performed with TimeScapes (version 1.2.2) [41]. TimeScapes employs a contact matrix built from distances between residues along with a median filter and Gaussian kernel to monitor the fraction of significant contact formation and breaking events per trajectory segment. For the current analysis, we used the same residue subset used for the PCA on crystal substructures (*i.e.* equivalent positions found in all crystal structures), plus the longer L5 and SI regions present in Eg5. Side chains were considered in contact if their distance was between 6.5 Å and 7.5 Å. The half width median filter was set to a value of 14 ns.

### Additional analysis of experimental and simulated structures

Complete-linkage hierarchical cluster analysis of the Euclidean distance matrix built from the projected structural coordinates along the first five eigenvectors was used to compare conformers. Note that a similar clustering pattern was observed when Cα atom RMSD was taken as the dissimilarity measure. Secondary structure analysis was performed with STRIDE [59] and VMD was used for molecular figure generation [60].

## Supporting Information

Figure S1 **Neck-linker state in relation to global motor domain conformation.** (**A**) Projection of all kinesin X-ray structures onto the principal planes defined by the two most significant PCs (PC1 and PC2). Structures are colored by neck-linker state, dark blue = fully docked, light blue = partially docked, lilac = undocked, gray = absent/unresolved. Partially docked indicates the presence of a hydrogen bond between N366 (NL) and G96 (α1b), I359 and N18-I19 (loop1), I299-L302-V303 (α4) and L324 (loop13); but with the C-terminal of the neck-linker (after N366) unresolved. Docked structures have additional contacts for positions following N366 and display a fully resolved C-terminal neck-linker segment. (**B**) Heat map clustering of kinesin structures in the PC1 to PC5 planes. Structure labels are colored by the neck linker state as in panel A. The dashed squares in the matrix and the dendogram lines correspond to the three ligand clusters in Figure 2.
(TIF)

Figure S2 **The contribution of each residue to the first two PCs.** Labels in the plot show the regions with the greatest contribution. Major elements of secondary structure are depicted with rectangles on the upper and lower axis (black for alpha helix, gray for beta strand).
(TIF)

Figure S3 **RMSD clustering of crystallographic structures.** The two cluster groups obtained from RMSD clustering are shown in the PC1–PC2 planes (red = ATP-like, green = ADP-like). See Figure 2 and main text for details.
(TIF)

Figure S4 **RMSD time series.** The temporal evolution of RMSD values from the initial structure for APO (**A**), ADP (**B**) and ATP (**C**) simulations. Replica runs are depicted with dash lines.
(TIF)

Figure S5 **Residue-wise RMSF values.** RMSF values are shown for APO (**A**), ADP (**B**) and ATP (**C**) simulations. Replica runs are depicted with dash lines.
(TIF)

Figure S6 **Comparison of crystallographic and aMD simulation derived principal components.** The average cumulative square inner product (ACSIP) between PC1–PC2 of the crystallographic dataset and eigenvectors derived from the combined APO (black), ADP (green) and ATP (red) simulations.
(TIF)

Figure S7 **Undocking of the neck-linker in simulations.** (**A**) Nucleotide free and (**B**) ATP bound Cβ-Cβ distances between I359 and L302 (blue), V303 (orange) and L324 (cyan). The dash line represents the cutoff for the interactions characteristic of docked neck liner regions in the crystallographic dataset. (**C–D**) Contact formation and breaking activity during nucleotide free and ATP bound simulations respectively. The plot reports the contact formation events (green), the contact breaking events (red) and total events (gray, formation + breaking) as a function of simulation time.
(TIF)

Figure S8 **Community composition across multiple cMD simulations.** Residues assigned to α4 (green), loop7 (yellow) and β4-β6-β7 (cyan) communities in eight independent 40 ns cMD simulations for (**A**) ATP and (**B**) ADP conditions.
(TIF)

Figure S9 **Results of PCA on kinesin structures with resolved neck-linker regions.** Conformer plot: projection of kinesin X-ray structures onto the principal planes defined by the two most significant PCs (PC1 and PC2). Structures are colored by ligand bound, triphosphate (red), diphosphate (green), and Eg5 inhibitor (pink). Structures are also labeled with their RCSB PDB code where space permits (see Table S1 for full details). Colored dashed ovals represent the major groupings obtained from hierarchical clustering of the projected structures in the PC1 to PC5 planes (see main text and Figure 1 for details). Insert: eigenvalue spectrum detailing results obtained from diagonalization of the atomic displacement correlation matrix of Ca atom coordinates. The magnitude of each eigenvalue is expressed as the percentage of the total variance (mean-square fluctuation) captured by the corresponding eigenvector. Labels beside each point indicate the cumulative sum of the total variance accounted for in all preceding eigenvectors.
(TIF)

Table S1 **Kinesin crystallographic structures.** The PDB codes of the kinesin family crystallographic structures analyzed are listed in column1. For each structure, the ligands present in the crystal unit cell are reported in column2, the nucleotide bound in column3 (red = ATP analogue, green = ADP, orange = ADP+Pi, gray = nucleotide free), the neck-linker conformation in column4 (dark blue = fully docked, light blue = partially docked, lilac = undocked, gray = absent). These colors are consistent with Figure S1.
(DOCX)

Table S2 **Selected time-averaged properties from simulations.**
(DOC)

Table S3 **Consistency of dynamic community partitioning in multiple cMD simulations.** Percentage of common residues for α4, loop7 and β4-β6-β7 communities for the 2 sets of eight 40 ns cMD simulations (see main text for details).
(DOC)

Table S4 **Critical nodes highlighted in ATP and ADP simulation sets.** List of residues with high betweeness values identified as critical for the connection of two communities in at least 4 cMD simulations. The residues identified as important for the communication between nucleotide-binding, microtubule-binding and neck-linker regions are highlighted in bold.
(DOC)
