# Supplementary figures and images for: Mapping the Structural and Dynamical Features of Kinesin Motor Domains
Source: PLoS Comput Biol. 2013 Nov 7;9(11):e1003329. doi: 10.1371/journal.pcbi.1003329 (PMC3820509; doi:10.1371/journal.pcbi.1003329)

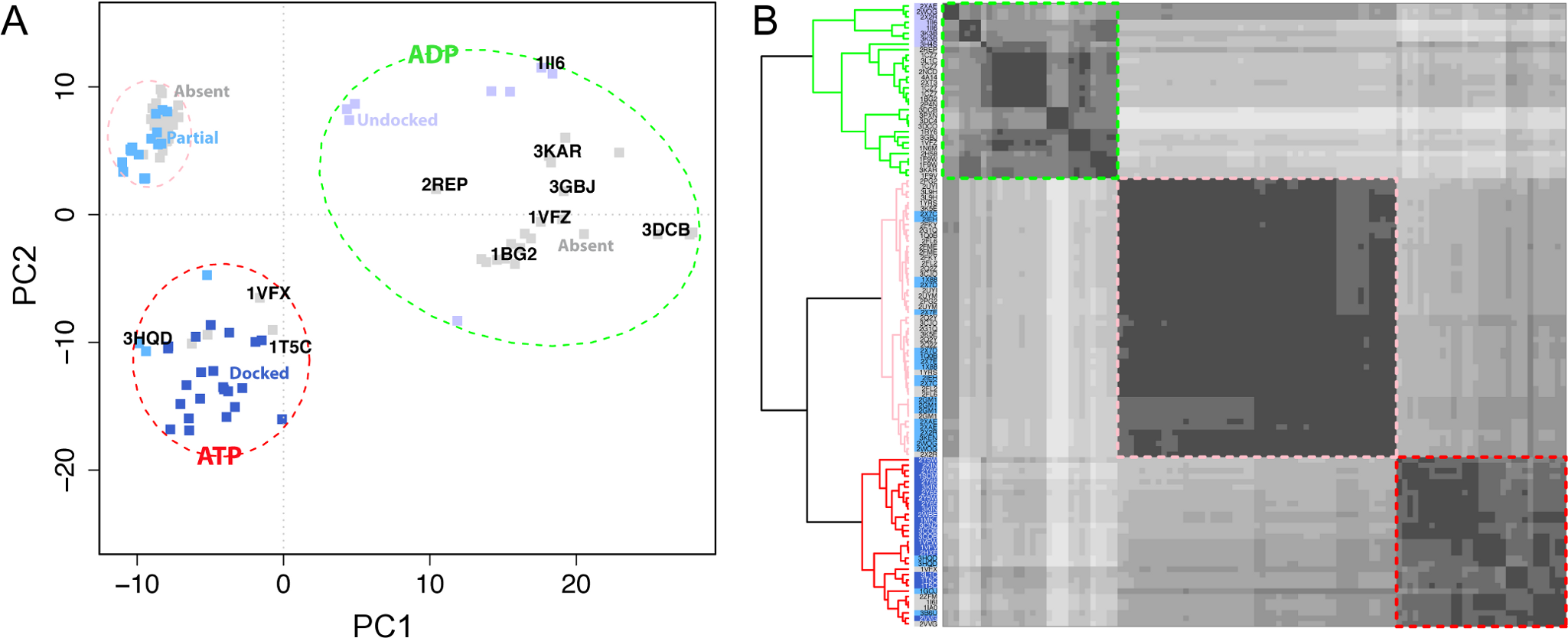

Supplement: Figure S1 — Neck-linker state in relation to global motor domain conformation. (A) Projection of all kinesin X-ray structures onto the principal planes defined by the two most significant PCs (PC1 and PC2). Structures are colored by neck-linker state, dark blue = fully docked, light blue = partially docked, lilac = undocked, gray = absent/unresolved. Partially docked indicates the presence of a hydrogen bond between N366 (NL) and G96 (α1b), I359 and N18-I19 (loop1), I299-L302-V303 (α4) and L324 (loop13); but with the C-terminal of the neck-linker (after N366) unresolved. Docked structures have additional contacts for positions following N366 and display a fully resolved C-terminal neck-linker segment. (B) Heat map clustering of kinesin structures in the PC1 to PC5 planes. Structure labels are colored by the neck linker state as in panel A. The dashed squares in the matrix and the dendogram lines correspond to the three ligand clusters in Figure 2. (TIF) [file pcbi.1003329.s001.tif]

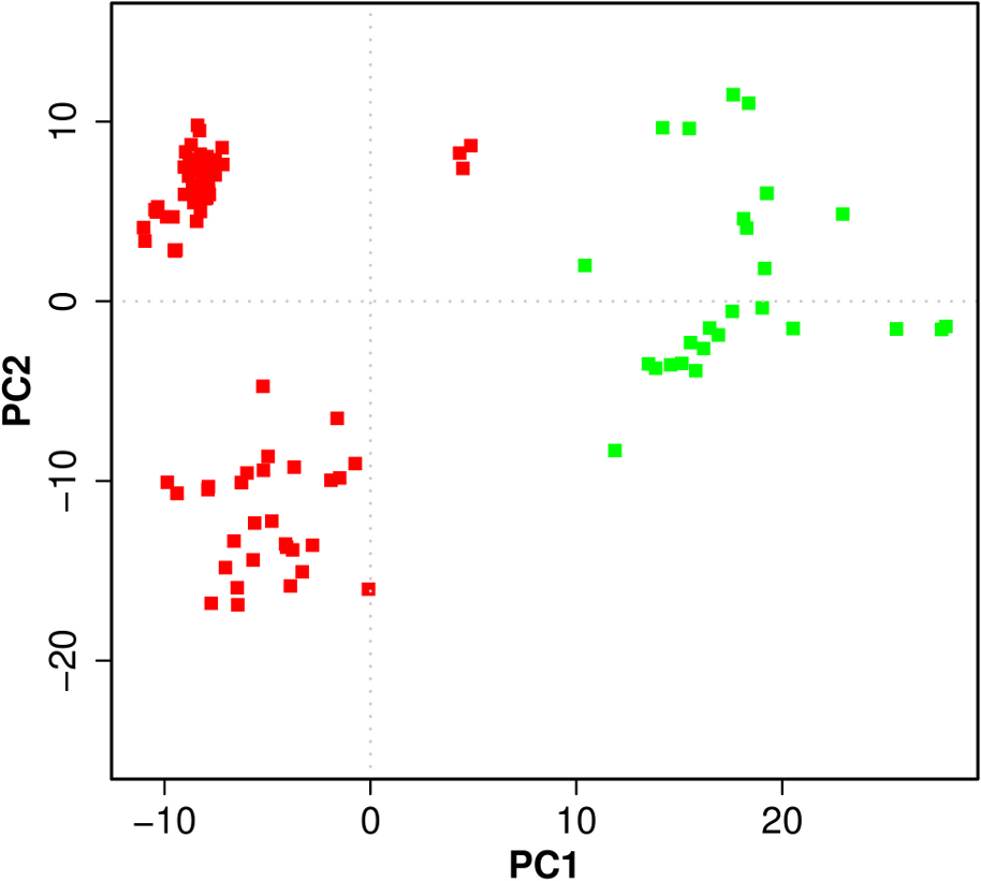

Supplement: Figure S3 — RMSD clustering of crystallographic structures. The two cluster groups obtained from RMSD clustering are shown in the PC1–PC2 planes (red = ATP-like, green = ADP-like). See Figure 2 and main text for details. (TIF) [file pcbi.1003329.s003.tif]

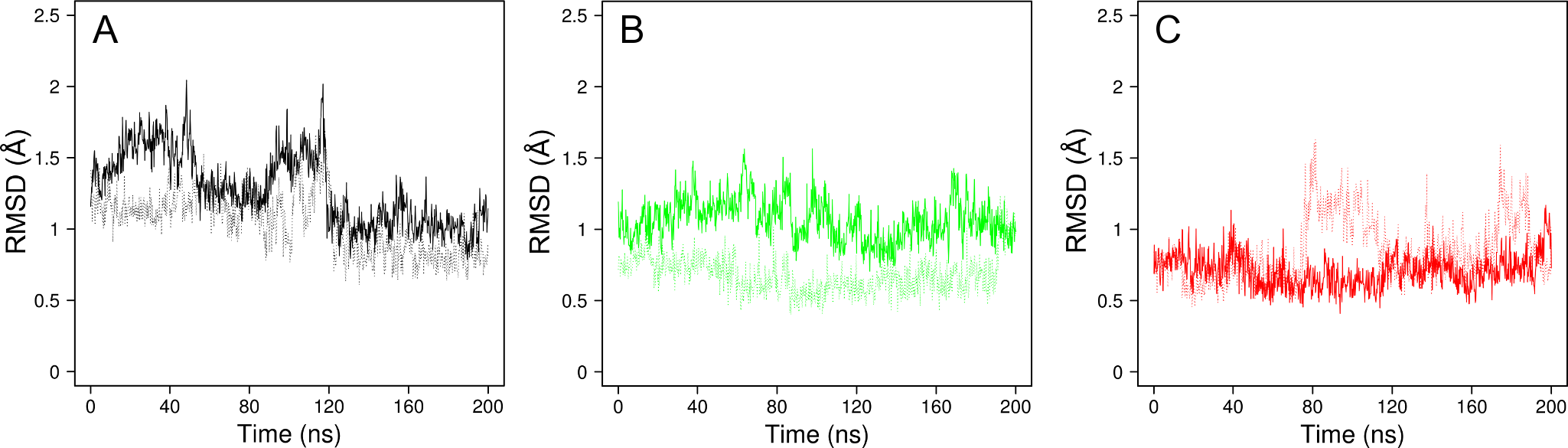

Supplement: Figure S4 — RMSD time series. The temporal evolution of RMSD values from the initial structure for APO (A), ADP (B) and ATP (C) simulations. Replica runs are depicted with dash lines. (TIF) [file pcbi.1003329.s004.tif]

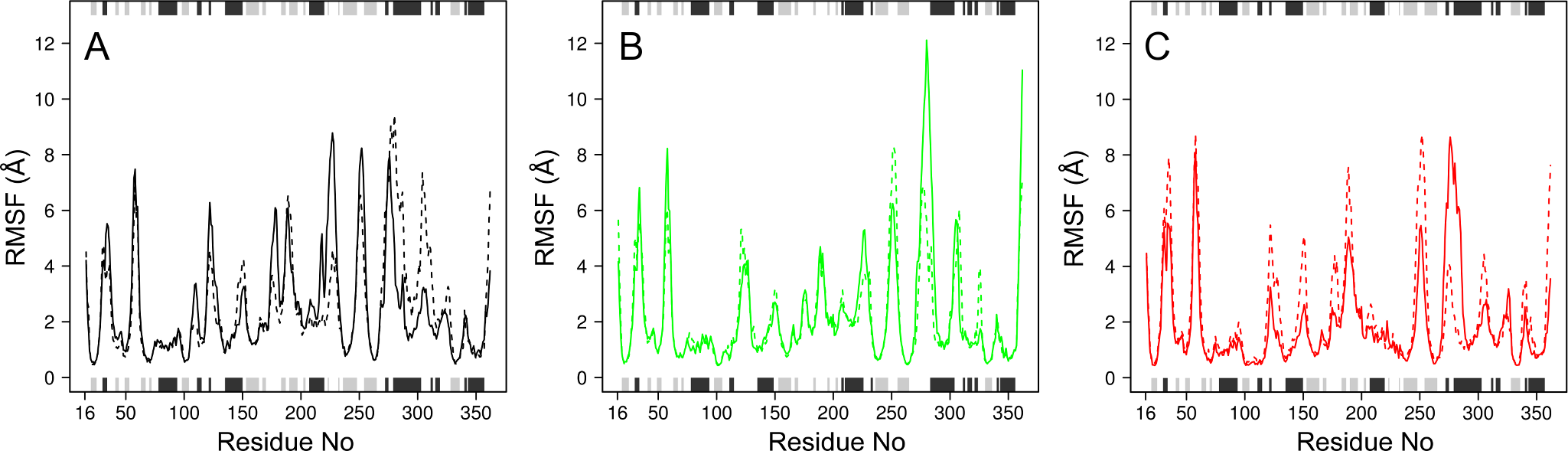

Supplement: Figure S5 — Residue-wise RMSF values. RMSF values are shown for APO (A), ADP (B) and ATP (C) simulations. Replica runs are depicted with dash lines. (TIF) [file pcbi.1003329.s005.tif]

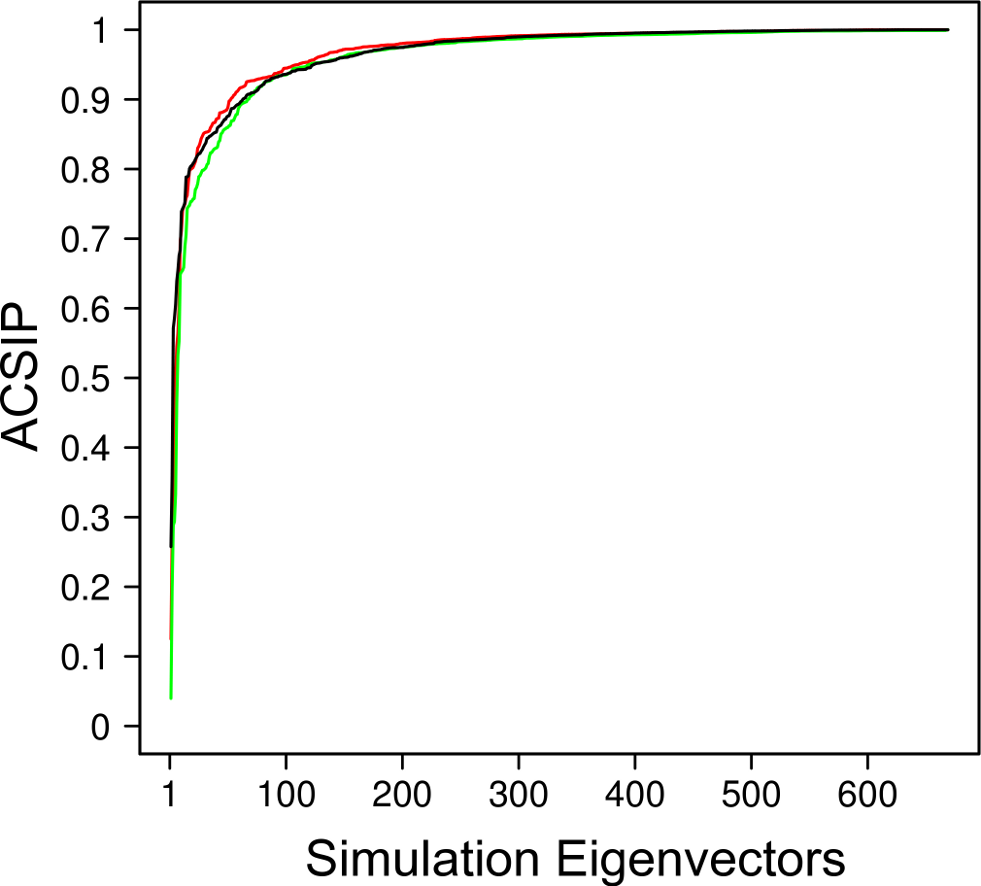

Supplement: Figure S6 — Comparison of crystallographic and aMD simulation derived principal components. The average cumulative square inner product (ACSIP) between PC1–PC2 of the crystallographic dataset and eigenvectors derived from the combined APO (black), ADP (green) and ATP (red) simulations. (TIF) [file pcbi.1003329.s006.tif]

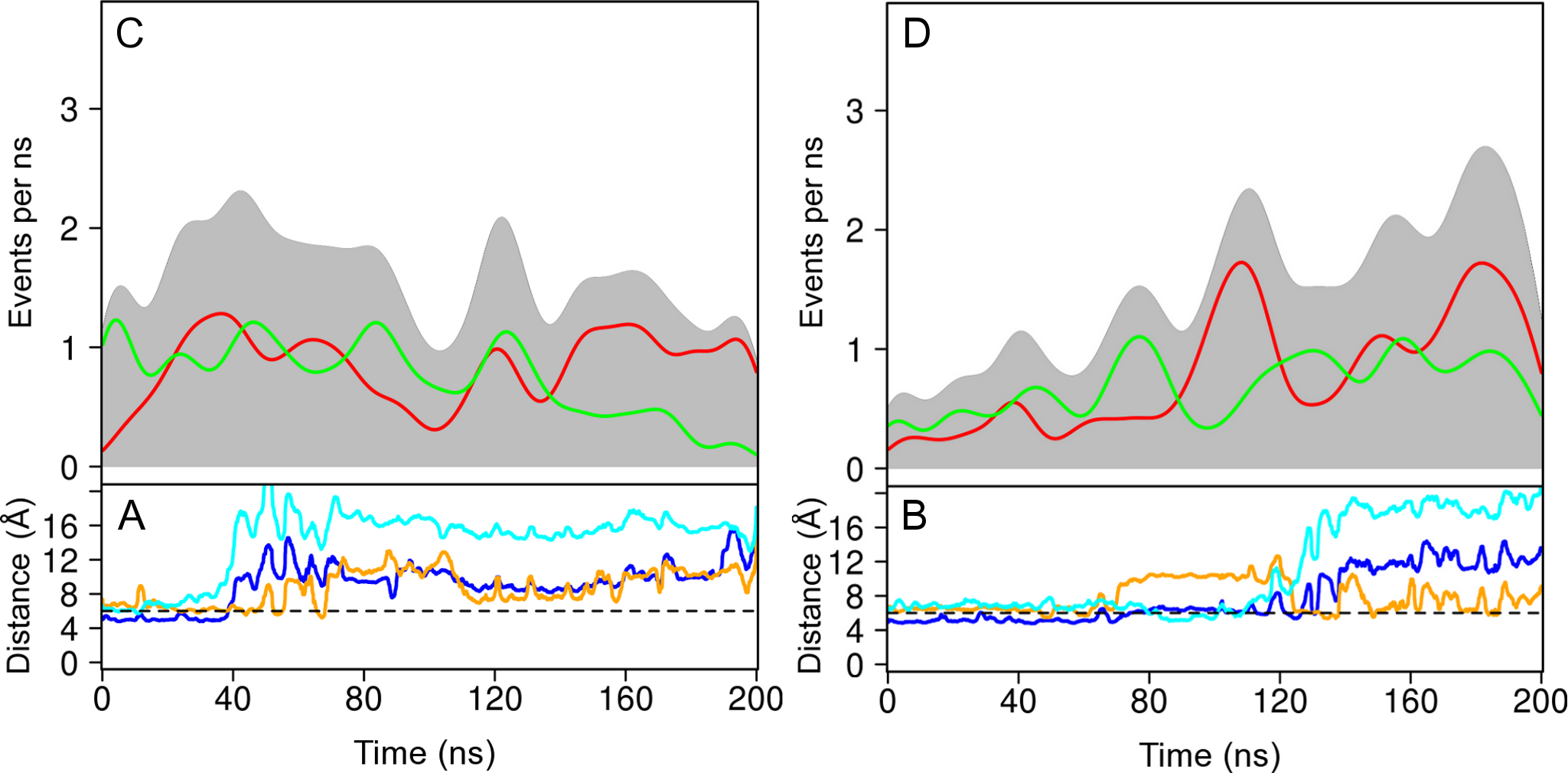

Supplement: Figure S7 — Undocking of the neck-linker in simulations. (A) Nucleotide free and (B) ATP bound Cβ-Cβ distances between I359 and L302 (blue), V303 (orange) and L324 (cyan). The dash line represents the cutoff for the interactions characteristic of docked neck liner regions in the crystallographic dataset. (C–D) Contact formation and breaking activity during nucleotide free and ATP bound simulations respectively. The plot reports the contact formation events (green), the contact breaking events (red) and total events (gray, formation + breaking) as a function of simulation time. (TIF) [file pcbi.1003329.s007.tif]

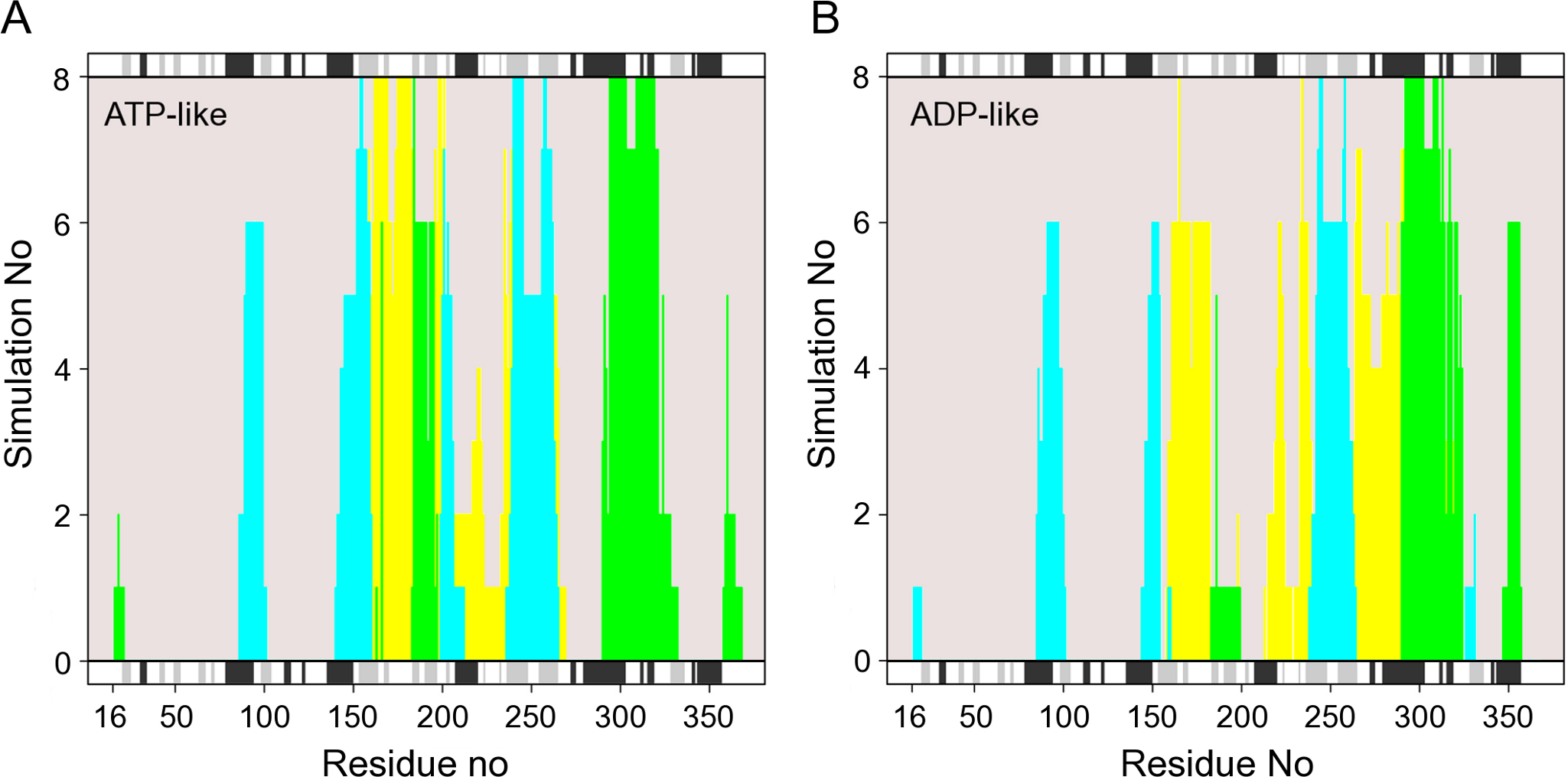

Supplement: Figure S8 — Community composition across multiple cMD simulations. Residues assigned to α4 (green), loop7 (yellow) and β4-β6-β7 (cyan) communities in eight independent 40 ns cMD simulations for (A) ATP and (B) ADP conditions. (TIF) [file pcbi.1003329.s008.tif]

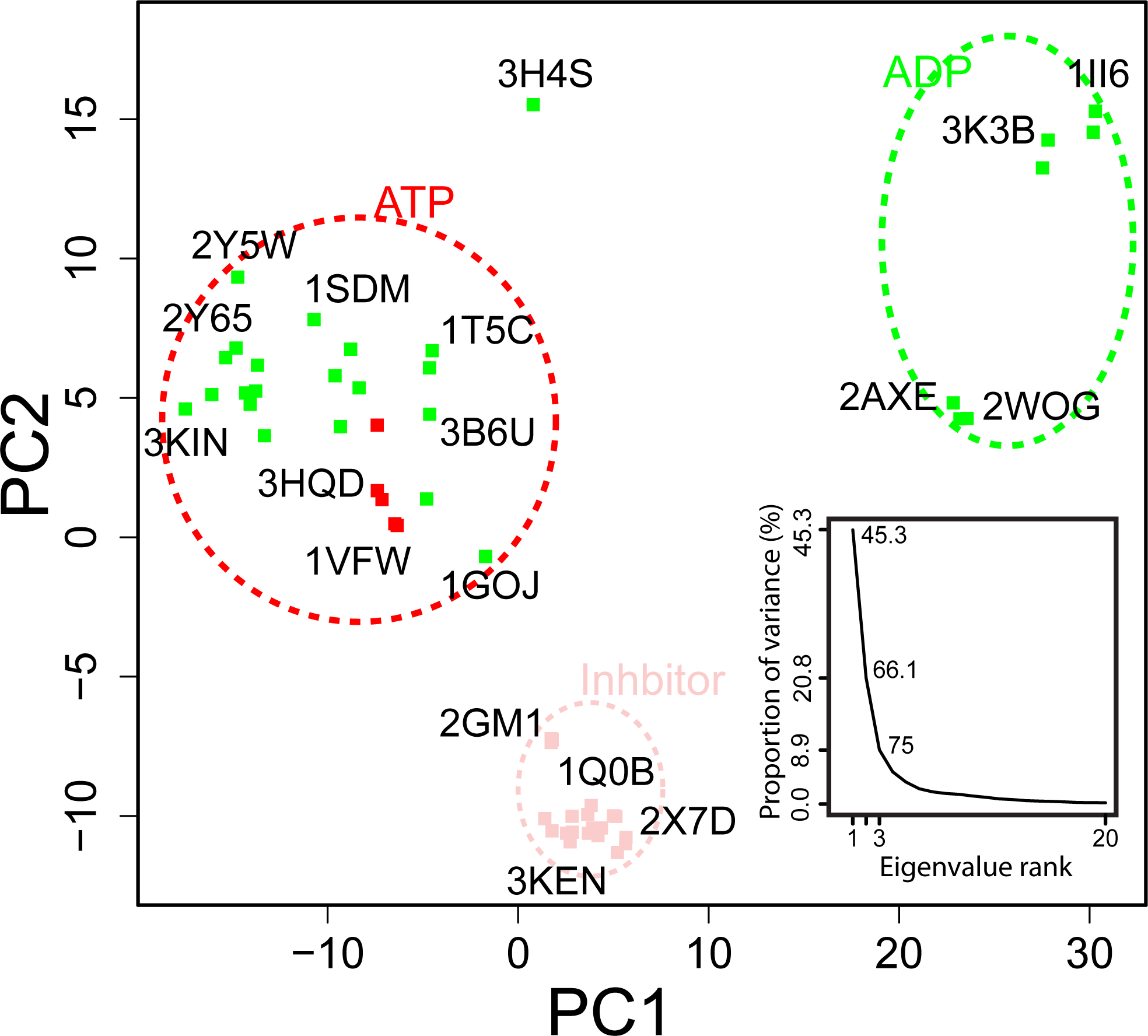

Supplement: Figure S9 — Results of PCA on kinesin structures with resolved neck-linker regions. Conformer plot: projection of kinesin X-ray structures onto the principal planes defined by the two most significant PCs (PC1 and PC2). Structures are colored by ligand bound, triphosphate (red), diphosphate (green), and Eg5 inhibitor (pink). Structures are also labeled with their RCSB PDB code where space permits (see Table S1 for full details). Colored dashed ovals represent the major groupings obtained from hierarchical clustering of the projected structures in the PC1 to PC5 planes (see main text and Figure 1 for details). Insert: eigenvalue spectrum detailing results obtained from diagonalization of the atomic displacement correlation matrix of Ca atom coordinates. The magnitude of each eigenvalue is expressed as the percentage of the total variance (mean-square fluctuation) captured by the corresponding eigenvector. Labels beside each point indicate the cumulative sum of the total variance accounted for in all preceding eigenvectors. (TIF) [file pcbi.1003329.s009.tif]
