# Supplementary material for: Mapping the Structural and Dynamical Features of Kinesin Motor Domains
Source: PLoS Comput Biol. 2013 Nov 7;9(11):e1003329. doi: 10.1371/journal.pcbi.1003329 (PMC3820509; doi:10.1371/journal.pcbi.1003329)
Supplement: Table S1 — Kinesin crystallographic structures. The PDB codes of the kinesin family crystallographic structures analyzed are listed in column1. For each structure, the ligands present in the crystal unit cell are reported in column2, the nucleotide bound in column3 (red = ATP analogue, green = ADP, orange = ADP+Pi, gray = nucleotide free), the neck-linker conformation in column4 (dark blue = fully docked, light blue = partially docked, lilac = undocked, gray = absent). These colors are consistent with Figure S1. (DOCX) [file pcbi.1003329.s010.docx]

| **PDB ID** | **Ligands** | **Nucleotide** | **Neck-Linker** | **Reference** |
| --- | --- | --- | --- | --- |
| 1RY6 | SO4 | NA | A | Shipley et al. Embo J. (2004) |
| 2ZFM | ADP | DP | A | Nitta et al. Nat.Struct.Mol.Biol. (2008) |
| 1BG2 | ACT, ADP, MG | DP | A | Kull et al. Nature (1996) |
| 1CZ7 | ADP, MG | DP | A | Kozielski et al. Structure Fold.Des. (1999) |
| 1F9V | ADP, MG | DP | A | Yun et al. EMBO J. (2001) |
| 1F9W | ADP, MG | DP | A | Yun et al. EMBO J. (2001) |
| 1GOJ | ADP, MG | DP | PD | Song et al. Embo J. (2001) |
| 1I6I | ACP, MG, TRS | TP | A | Kikkawa et al. Nature (2001) |
| 1IA0 | GTP, GDP, TXL, ACP, MG | TP | A | Kikkawa et al. Nature (2001) |
| 1II6 | ADP, MG, NO3 | DP | U | Turner et al. J.Biol.Chem. (2001) |
| 1MKJ | ADP, MG, SO4 | DP | FD | Sindelar et al. Nat.Struct.Mol.Biol. (2002) |
| 1N6M | ADP, MG | DP | A | Yun et al. EMBO J. (2003) |
| 1Q0B | ADP, MG, NAT | DP | Chain A: PD, | Yan et al. J.Mol.Biol. (2004) |
|  |  |  | Chain B: A |  |
| 1SDM | ADP, MG | DP | FD | Vinogradova et al. J.Biol.Chem. (2004) |
| 1T5C | ADP, MG, NO3, PIN | DP | FD | Garcia-Saez et al. J.Mol.Biol. (2004) |
| 1VFV | ANP, MG | TP | FD | Nitta et al. Science (2004) |
| 1VFW | ANP, MG | TP | FD | Nitta et al. Science (2004) |
| 1VFX | ADP, AF3, MG, TRS | DP TP | A | Nitta et al. Science (2004) |
| 1VFZ | ADP, MG, VO4 | DP TP | A | Nitta et al. Science (2004) |
| 1X88 | ADP, MG, NAT | DP | PD | Maliga et al. TO BE PUBLISHED (NA) |
| 1YRS | ADP, L47, MG | DP | A | Cox et al. BIOORG.MED.CHEM.LETT. (2005) |
| 2FKY | ADP, MG, N2T | DP | A | Fraley et al. Bioorg.Med.Chem.Lett. (2006) |
| 2FL2 | ADP, MG, N4T | DP | A | Fraley et al. Bioorg.Med.Chem.Lett. (2006) |
| 2FL6 | ADP, MG, N5T | DP | A | Fraley et al. Bioorg.Med.Chem.Lett. (2006) |
| 2FME | 3QC, ADP, MG | DP | A | Tarby et al. Bioorg.Med.Chem.Lett. (2006) |
| 2G1Q | ADP, MG, N9H | DP | A | Cox et al. Bioorg.Med.Chem.Lett. (2006) |
| 2GM1 | 2AZ, ADP, MG | DP | Chains: A-C-D PD, | Kim et al. Bioorg.Med.Chem.Lett. (2006) |
|  |  |  | Chain B: A |  |
| 2H58 | ADP, MG, UNX | DP | A | Wang et al. To be Published (NA) |
| 2HXF | GTP, MG, GDP, TA1, ANP | TP | FD | Kikkawa et al. Embo J. (2006) |
| 2IEH | ADP, CL, K, MG, MOY, PG4 | DP | PD | Garcia-Saez et al. J.Biol.Chem. (2007) |
| 2KIN | ADP, SO4, | DP | FD | Sack et al. Biochemistry (1997) |
| 2NCD | ADP, SO4 | DP | A | Sablin et al. Nature (1998) |
| 2P4N | MG, ZN, GDP, GTP, | DP | A | Sindelar et al. J.Cell Biol. (2007) |
|  | TA1, ADP |  |  |  |
| 2PG2 | ADP, K01, MG | DP | A | Pinkerton et al. Bioorg.Med.Chem.Lett. (2007) |
| 2Q2Y | ADP, MG, MKR | DP | A | Roecker et al. Bioorg.Med.Chem.Lett. (2007) |
| 2Q2Z | ADP, MG, MKK | DP | A | Roecker et al. Bioorg.Med.Chem.Lett. (2007) |
| 2REP | ADP, MG, UNX | DP | A | Zhu et al. To be Published (NA) |
| 2UYI | ADP, K02, MG | DP | A | Pinkerton et al. Bioorg.Med.Chem.Lett. (2007) |
| 2UYM | ADP, K03, MG | DP | A | Pinkerton et al. Bioorg.Med.Chem.Lett. (2007) |
| 2VVG | ADP, MG | DP | FD | Hoeng et al. Mol.Biol.Cell (2008) |
| 2WBE | MG, GDP, TA1, ANP, GTP | TP | FD | Bodey et al. J.Mol.Biol. (2009) |
| 2WOG | ADP, MG, ZZD | DP | Chains A-B: PD, | Kaan et al. Biochem.J. (2010) |
|  |  |  | Chain C: U |  |
|  |  |  |  |  |
| 2X2R | ADP, MG, X2O | DP | Chain A: PD, | Kaan et al. J.Med.Chem. (2011) |
|  |  |  | Chain B: A, Chain C: U |  |
| 2X7C | ADP, KZ9, MG | DP | PD | Kaan et al. J.Med.Chem. (2010) |
| 2X7D | ADP, EGB, MG | DP | PD | Kaan et al. J.Med.Chem. (2010) |
| 2X7E | ADP, MG, X7E | DP | PD | Kaan et al. J.Med.Chem. (2010) |
| 2XAE | 2XA, ADP, CL, MG, SO4 | DP | Chains A-B: PD, | Kaan et al. J.Med.Chem. (2011) |
|  |  |  | Chain C: U |  |
| 2XT3 | ADP, MG | DP | A | Klejnot et al. Acta Crystallogr.,Sect.D (2012) |
| 2Y5W | ADP, MG | DP | FD | Kaan et al. Science (2011) |
| 2Y65 | ADP, MG, | DP | FD | Kaan et al. Science (2011) |
| 3B6U | ADP, MG, UNX | DP | FD | Zhu et al. To be Published (NA) |
| 3CJO | ADP, K30, MG | DP | A | Cox et al. J.Med.Chem. (2008) |
| 3CNZ | ADP, MG | DP | FD | Vinogradova et al. J.Struct.Biol. (2008) |
| 3COB | ADP, MG | DP | FD | Vinogradova et al. J.Struct.Biol. (2008) |
| 3DC4 | ADP, MG | DP | A | Cochran et al. Cell (2009) |
| 3DCB | ANP, MG | TP | A | Cochran et al. Cell (2009) |
| 3DCO | MG, ZN, GDP, GTP, | DP | A | Cochran et al. Cell (2009) |
|  | TA1, ADP |  |  |  |
| 3GBJ | ADP, MG, UNX | DP | A | Tong et al. To be Published (NA) |
| 3H4S | ADP, MG, CA | DP | U | Vinogradova et al. Proc.Natl.Acad.Sci.USA |
|  |  |  |  | (2009) |
| 3HQD | ANP, MG, PO4 | TP | PD | Parke et al. J.Biol.Chem. (2010) |
| 3K3B | ADP, CL, L31, MG, NO3, PEG | DP | U | Barsanti et al. Bioorg.Med.Chem.Lett. (2010) |
| 3K5E | ADP, K5E, MG | DP | A | Crawley et al. To be Published (NA) |
| 3KAR | ADP, MG | DP | A | Gulick et al. Biochemistry (1998) |
| 3KEN | ADP, KEN, MG, ZZD | DP | PD | Kim et al. J.Biol.Chem. (2010) |
| 3KIN | ADP, | DP | FD | Kozielski et al. Cell (1997) |
| 3L1C | ADP, MG | DP | A | Heuston et al. Bmc Struct.Biol. (2010) |
| 3L9H | ADP, EMQ | DP | A | Schiemann et al. Bioorg.Med.Chem.Lett. |
|  |  |  |  | (2010) |
| 3PXN | ADP, MN | DP | A | Cochran et al. To be Published (NA) |
| 4A14 | ADP, MG | DP | A | Klejnot et al. Acta Crystallogr.,Sect.D (2012) |
