# Supplementary material for: Mapping the Structural and Dynamical Features of Kinesin Motor Domains
Source: PLoS Comput Biol. 2013 Nov 7;9(11):e1003329. doi: 10.1371/journal.pcbi.1003329 (PMC3820509; doi:10.1371/journal.pcbi.1003329)
Supplement: Table S2 — Selected time-averaged properties from simulations. (DOC) [file pcbi.1003329.s011.doc]

|  | **Radius of gyration (Å)** | **Beta strand content (%)** | **Alpha helix content (%)** | **Core residue RMSD (Å)** |
| --- | --- | --- | --- | --- |
| **APO-1** | 17.52 ± 0.13 | 25 ± 1 | 24 ± 4 | 1.22 ± 0.2 |
| **APO-2** | 17.50 ± 0.09 | 27 ± 1 | 25 ± 3 | 1.00 ± 0.2 |
| **ADP-1** | 17.37 ± 0.09 | 25 ± 1 | 26 ± 3 | 0.77 ± 0.1 |
| **ADP-2** | 17.47 ± 0.10 | 25 ± 1 | 27 ± 2 | 0.75 ± 0.2 |
| **ATP-1** | 17.42 ± 0.09 | 27 ± 1 | 27 ± 2 | 0.70 ± 0.1 |
| **ATP-2** | 17.42 ± 0.01 | 25 ± 1 | 27 ± 2 | 0.86 ± 0.2 |
| **Xray ensemble** | 17.10 ± 0.11 | 24 ± 2 | 30 ± 3 | 0.40 ± 0.1 |
