# Supplementary material for: Mapping the Structural and Dynamical Features of Kinesin Motor Domains
Source: PLoS Comput Biol. 2013 Nov 7;9(11):e1003329. doi: 10.1371/journal.pcbi.1003329 (PMC3820509; doi:10.1371/journal.pcbi.1003329)
Supplement: Table S3 — Consistency of dynamic community partitioning in multiple cMD simulations. Percentage of common residues for α4, loop7 and β4-β6-β7 communities for the 2 sets of eight 40 ns cMD simulations (see main text for details). (DOC) [file pcbi.1003329.s012.doc]

| Percentage of identical residues among communities from ATP-like rMD and aMD simulations (%) | | | |
| --- | --- | --- | --- |
|  | **aMD-ATP like**  **(α4 community)** | **aMD-ATP like**  **(loop7 community)** | **aMD-ATP like**  **(β6-β7 community)** |
| **rMD1-ATP-like** | 90 | 72 | 36 |
| **rMD2-ATP-like** | 60 | 64 | 80 |
| **rMD3-ATP-like** | 60 | 81 | 93 |
| **rMD4-ATP-like** | 76 | 38 | 55 |
| **rMD5-ATP-like** | 92 | 45 | 62 |
| **rMD6-ATP-like** | 82 | 29 | 73 |
| **rMD7-ATP-like** | 78 | 59 | 87 |
| **rMD8-ATP-like** | 80 | 53 | 49 |
| Percentage of identical residues among communities from ADP-like rMD and aMD simulations (%) | | | |
|  | **aMD-ADP like**  **(α4 community)** | **aMD-ADP like**  **(loop7 community)** | **aMD-ADP like**  **(β6-β7 community)** |
| **rMD1-ADP-like** | 86 | 78 | 100 |
| **rMD2-ADP-like** | 70 | 82 | 65 |
| **rMD3-ADP-like** | 62 | 44 | 100 |
| **rMD4-ADP-like** | 92 | 53 | 52 |
| **rMD5-ADP-like** | 92 | 42 | 70 |
| **rMD6-ADP-like** | 92 | 62 | 100 |
| **rMD7-ADP-like** | 92 | 53 | 100 |
| **rMD8-ADP-like** | 62 | 53 | 49 |
