# Supplementary material for: Mapping the Structural and Dynamical Features of Kinesin Motor Domains
Source: PLoS Comput Biol. 2013 Nov 7;9(11):e1003329. doi: 10.1371/journal.pcbi.1003329 (PMC3820509; doi:10.1371/journal.pcbi.1003329)
Supplement: Table S4 — Critical nodes highlighted in ATP and ADP simulation sets. List of residues with high betweeness values identified as critical for the connection of two communities in at least 4 cMD simulations. The residues identified as important for the communication between nucleotide-binding, microtubule-binding and neck-linker regions are highlighted in bold. (DOC) [file pcbi.1003329.s013.doc]

| **Critical nodes obtained from cMD simulations** | |
| --- | --- |
| **ATP-like** | **ADP-like** |
| K17 | H38 |
| I19 | V71 |
| M70 | F72 |
| V71 | S76 |
| A74 | K77 |
| T76 | R83 |
| E92 | C87 |
| C99 | C99 |
| T100 | T100 |
| Q106 | F102 |
| F144 | A103 |
| S159 | T112 |
| L160 | I143 |
| L161 | V158 |
| **Y164** | L160 |
| **E166** | L161 |
| **R221** | L182 |
| **R234** | Q183 |
| S235 | L199 |
| **E284** | E200 |
| T294 | M228 |
| I299 | Y231 |
| E345 | S232 |
| **I359** | S233 |
|  | T242 |
|  | N289 |
|  | Q290 |
|  | S323 |
|  | L324 |
|  | T328 |
|  | I332 |
|  | I333 |
|  | T349 |
|  | K357 |
